# Copper-zirconia interfaces in UiO-66 enable selective catalytic hydrogenation of CO_2_ to methanol

**DOI:** 10.1038/s41467-020-19438-w

**Published:** 2020-11-18

**Authors:** Yifeng Zhu, Jian Zheng, Jingyun Ye, Yanran Cui, Katherine Koh, Libor Kovarik, Donald M. Camaioni, John L. Fulton, Donald G. Truhlar, Matthew Neurock, Christopher J. Cramer, Oliver Y. Gutiérrez, Johannes A. Lercher

**Affiliations:** 1 Institute for Integrated Catalysis, and Physical and Computational Sciences Directorate, Pacific Northwest National Laboratory, Richland, WA 99354 USA; 2 Department of Chemistry, Minnesota Supercomputing Institute, Chemical Theory Center, and Inorganometallic Catalyst Design Center, University of Minnesota, Minneapolis, MN 55455 USA; 3 William R. Wiley Environmental Molecular Sciences Laboratory, Pacific Northwest National Laboratory, Richland, WA 99354 USA; 4 Present Address: Department of Chemistry and Biomolecular Science, Clarkson University, Potsdam, NY 13699 USA

**Keywords:** Catalyst synthesis, Heterogeneous catalysis, Metal-organic frameworks

## Abstract

Molecular interactions with both oxides and metals are essential for heterogenous catalysis, leading to remarkable synergistic impacts on activity and selectivity. Here, we show that the direct link between the two phases (and not merely being together) is required to selectively hydrogenate CO_2_ to methanol on catalysts containing Cu and ZrO_2_. Materials consisting of isolated Cu particles or atomically dispersed Cu–O–Zr sites only catalyze the reverse water-gas shift reaction. In contrast, a metal organic framework structure (UiO-66) with Cu nanoparticles occupying missing-linker defects maximizes the fraction of metallic Cu interfaced to ZrO_2_ nodes leading to a material with high adsorption capacity for CO_2_ and high activity and selectivity for low-temperature methanol synthesis.

## Abstract (web summary)

Molecular interactions with both oxides and metals are essential for heterogenous catalysis, leading to remarkable impacts on activity. Here the authors show that a direct link between Cu and ZrO2 in a metal organic framework is required to hydrogenate CO2 to methanol.

## Introduction

Phase boundaries at metal–metal oxide interfaces induce unique electronic structures as well as specific substrate binding, reactivity, and heterogeneous catalytic activity. Such interface sites have been hypothesized to enable activation and selective hydrogenation of CO and CO_2_^1–5^. An adjacent metal phase is required to catalyze H_2_ dissociation and to provide hydrogen atoms at a sufficient rate for reduction of CO_*x*_^1–3,6–8^. Cu supported on ZrO_2_ has attracted attention for its high selectivity for the conversion of CO_2_ to methanol, which has been hypothesized to be related to intermediates at the metal–metal oxide interfaces^3,9,10^. It should be noted in passing that oxides such as ZnO and ZrO_2_ also are able to dissociate H_2_, albeit via a heterolytic path and at a slow rate^11^.

While a large variety of successful catalytic systems have been reported^12^, stable development of a next-generation methanol synthesis catalyst that provides greater flexibility with respect to the operational thermal window requires addressing two main aspects. The first is related to the question whether a direct chemical link between the oxide support and the metal is required or whether the mere intimate presence of the two phases at molecular distances is sufficient. The second aspect is related to the question of whether a single Cu atom suffices to catalyze hydrogenation or whether an extended metal particle is required to enable a sufficient supply rate of atomic hydrogen.

To address these questions, we decided not to use conventional anchoring of Cu on a ZrO_2_ support (Supplementary Fig. 1a) because the inherent diversity of the oxide surface and the irregular porosity does not allow controlled variation of the interface and the metal nuclearity. Instead, we used the atomically precise oxide node of a metal organic framework (MOF) as a support for the metal particles and their pores to ensure molecular-level proximity, even when stable chemical links were not established (Supplementary Fig. 1b). The MOF UiO-66 with partly under-coordinated oxide nodes containing six Zr cations is used as a support for Cu clusters of varying nuclearity. The porous structure of the MOF beneficially enhances the proximity of the oxide node to the metal particle, even in the absence of a direct link. We modified the synthesis procedure and Cu loading to obtain a series of catalysts with varying particle size of Cu and chemical interaction with the zirconia (ZrO_2_) nodes. The performances of the materials in the hydrogenation of CO_2_ to methanol highlight the potential of taking advantage of the MOF structure to attain a precise control of active site structure and catalytic pathways.

## Results

### Synthesis of interacting and non-interacting Cu species in UiO-66

Two methods have been reported for the synthesis of Cu particles into MOF pores, one relying on synthesizing a MOF structure around preformed Cu particles^13^ and the other relying on covalently binding Cu–ZnO composites to linkers through pyridine functional groups^4^; see Supplementary Fig. 1c, routes a and b. Neither of these two methods would allow specific homotopic anchoring Cu at the node. To overcome this limitation, we exchanged Cu^2+^ cations^14^ for protons at under-coordinated Zr_6_O_8_ nodes of UiO-66 (Supplementary Fig. 1c, route c)^15^ that upon reduction at mild temperatures form metallic Cu particles, while not breaking a large fraction of the Cu–O–Zr bonds. By varying the loading and the reduction procedure, single Cu^δ+^ atoms and Cu particles not in contact with the node were synthesized also.

**Fig. 1:**
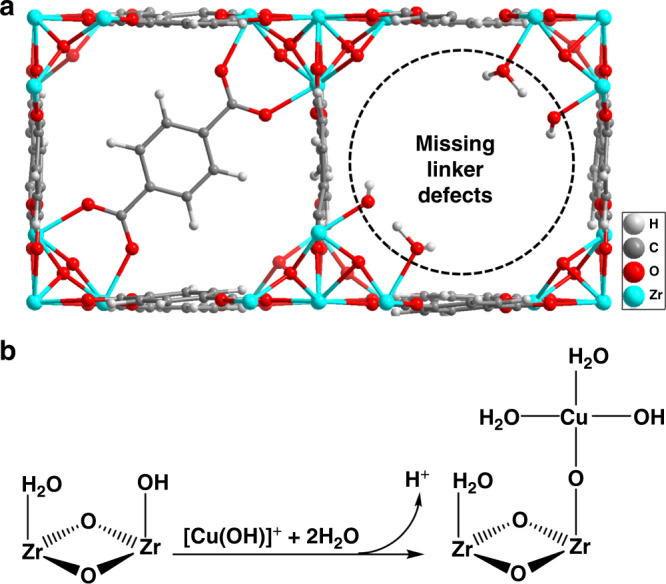
Using defective nodes to deposit Cu species. **a** A linker missing in the UiO-66 structure is replaced by two –OH/OH_2_ species, which are active for ion exchange to deposit metal onto the Zr_6_ nodes of the MOF. **b** Possible reaction for Cu deposition (ion exchange) in aqueous solution.

For a MOF such as UiO-66, coordination of the Zr_6_O_8_ nodes requires eliminating a controlled concentration of linkers (creating under-coordinated sites)^16^ to form a local environment that can host Cu particles. We synthesized such under-coordinated UiO-66 by using dimethylformamide as a solvent at 80 °C; this selectively leads to –OH and/or –OH_2_ groups that replace a fraction of the linkers^17^. The presence of hydroxyl groups was confirmed by infrared (IR) spectroscopy for the series of prepared materials (Supplementary Fig. 2). Using the differences in the pore volume between the as-completely-coordinated-as-possible reference UiO-66 and the UiO-66 with under-coordinated nodes, we estimate that one out of six carboxylate linkers per Zr_6_O_8_ unit was missing and, hence, two additional OH groups per node (Fig. 1a)^16–19^.

A series of catalyst precursors with varying Cu concentrations was prepared by exchanging the hydroxyl groups at the Zr_6_O_8_ nodes with aqua hydroxo Cu cations by putting the materials on contact with solutions containing Cu(OH)^+^ at pH 5 (Fig. 1b)^14^. The material with the optimum Cu loading of 1.4 wt.% was named in its final form Cu/UiO-66-a. The Cu contents (0.4 Cu atoms per Zr_6_O_8_ node) were commensurate with the decrease in concentration of OH groups determined from the corresponding IR spectra (~43% based on the integrated area of the IR band in Supplementary Fig. 2). Subsequently, the catalyst precursors were reduced in 25 vol.% H_2_/N_2_ at 200 °C, causing reduction of Cu^2+^ and formation of metallic particles.

A second type of Cu catalyst, identical in Cu loading and particle size to Cu/UiO-66-a (i.e., 1.4 wt.% Cu [~0.4 Cu atoms per node] and 0.7 nm) was prepared and named Cu/UiO-66-b. We synthesized this material by impregnating UiO-66 with an aqueous solution of Cu(NO_3_)_2_. Despite the introduction of similar Cu content (1.8 wt.%) as that in Cu/UiO-66-a, the decrease in concentration of OH groups was only 18%. This indicates less specific interaction between Cu^2+^ species and the MOF structure. In both materials (Cu/UiO-66-a and Cu/UiO-66-b), Cu is introduced in the pores, and the decrease in pore volume upon Cu incorporation was the same, whereas the shape of the N_2_-sorption isotherms and the X-ray diffraction patterns characteristic of UiO-66 were preserved upon modification (Supplementary Figs. 3 and 4). The chemical and structural properties of these materials are provided in Table 1.

**Table 1:** Textural properties of selected materials, level of –OH groups exchanged, Cu content in the modified MOFs, and dispersion of the Cu species.

Catalysts	Specific surface area (m^2^/g)^a^	Pore volume (cm^3^/g)^a^	Level of –OH exchanged by Cu (%)^b^	Cu loading (wt. %)^c^	Dispersion (%)^d^
UiO-66	1550	0.63	-	-	-
Cu/UiO-66-a	1468	0.61	43.2	1.40	85
Cu/UiO66-b	1432	0.60	18.3	1.80	84

^a^Determined by N_2_-sorption measurements.

^b^Determined by IR spectroscopy.

^c^Measured by inductively coupled plasma optical emission spectrometry.

^d^Calculated based on the coordination number of the first Cu–Cu shell derived from extended X-ray absorption fine structure (EXAFS) fitting.

### Structure and chemical environment of interacting and non-interacting Cu species in UiO-66

As will be shown below via the catalytic results, it is critical to understand the nature of the Cu–ZrO_2_ interaction qualitatively and quantitatively. Cu K-edge X-ray absorption near edge structure (XANES) and extended X-ray absorption fine structure (EXAFS) were used to characterize the location and nature of the Cu particles in Cu/UiO-66-a and b, which are hypothesized to differ with respect to the specific binding between the Cu metal particles and the ZrO_2_ nodes. A detailed analysis of the nuclearity of Cu species and their interactions with the nodes is given in the Supplementary Note. In the following discussion, we report the key results and conclusions from our analyses and support our conclusions with information from electron microscopy characterizations.

We use the position and intensity of the white line (8978 eV) in Cu K-edge XANES to characterize the average environment and structure of Cu particles. For Cu/UiO-66-a, the XANES data indicate that there is a distribution of oxidation estates of Cu (Supplementary Fig. 5 and Supplementary Table 1). In particular, 66% of Cu atoms are metallic and approximately 34% of Cu atoms are cationic.

The comparison among Fourier-transformed imaginary χ(R) EXAFS spectra of Cu/UiO-66-a with different *k*^*n*^ weightings allows differentiation of low-Z and high-Z scattering elements around Cu because scattering contributions from elements with higher mass increase with *k* weighting^20–22^. The feature at approximately 0.3 nm, which was not present in the EXAFS spectra of the Cu foil reference, intensified as the *k* weighting increased (Supplementary Fig. 6). Thus, we conclude that the contribution stems from scattering by a nearby heavier element than Cu (i.e., Zr).

**Fig. 2:**
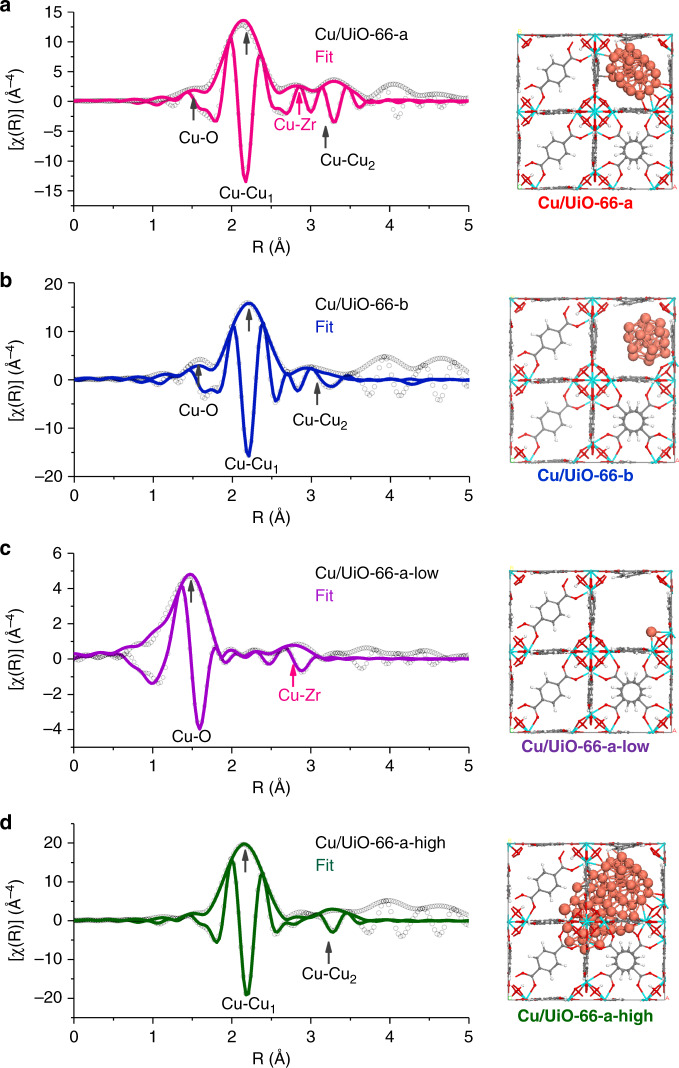
Structural models of selected materials. Cu K-edge *k*^3^-weighted EXAFS and Fourier-transform spectra with fitting lines of Cu/UiO-66-a, Cu/UiO-66-b, Cu/UiO-66-a-low, and Cu/UiO-66-a-high for which the Cu–Zr path is highlighted with red arrows. The figures on the right side of each row are schematic structural models deduced from the EXAFS analyses. They show the interface bonding of sub-nanometer Cu clusters with Zr_6_O_8_ nodes for Cu/UiO-66-a (**a**), the non-proximity of Cu clusters with Zr_6_ nodes for Cu/UiO-66-b (**b**), the bonding of isolated Cu with Zr_6_ nodes for Cu/UiO-66-a-low (**c**) and the higher fraction of metallic Cu species for Cu/UiO-66-a-high (**d**, where the Cu–O and Cu–Zr paths are negligible for the EXAFS fitting).

The EXAFS spectra were fitted to determine the average structural and chemical environment of Cu species. Figure 2a shows the results in both amplitude and imaginary parts. The prominent path at 0.22 nm in Fig. 2a is attributed to backscattering of the closest Cu–Cu coordination (denoted as Cu–Cu_1_). The features at distances larger than 0.33 nm are attributed to the backscattering Cu–Cu paths of higher shells (denoted as Cu–Cu_2_) that occur in Cu particles. Along the Cu–Cu backscattering paths, the features observed at 0.15 nm and 0.28 nm (not phase corrected) are attributed to the Cu–O and Cu–Zr paths, respectively (Supplementary Fig. 7). The EXAFS fitting parameters are listed in Supplementary Table 2, and the corresponding EXAFS oscillations, which agree well with experiment, are shown in Supplementary Fig. 8. Assuming the sub-nanometer Cu clusters in the cage of UiO-66 are nearly spherical, the average coordination number derived from the Cu–Cu_1_ path is ~6.5 ± 0.3. This allowed us to determine that the particle contains 25 ± 4 Cu atoms^23^. Such number of atoms is equivalent to an average diameter of 0.7–0.8 nm and a fraction of exposed Cu of 0.85 (see Supplementary Figs. 9 and 10 for the calculations and Fig. 2a for the optimized geometry). The calculated coordination numbers of Cu–O and Cu–Zr were 0.3–0.4, indicating that approximately 30% of the Cu atoms are bonded to the Zr_6_O_8_ nodes via oxygen bridges (Fig. 2a). This is in excellent agreement with the fraction of Cu atoms with cationic character estimated by XANES analysis. Thus, we conclude that the Cu particles in Cu/UiO-66-a are covalently bonded to ZrO_2_ nodes via Cu–O–Zr sites, wherein Cu has a positive charge.

We carried out density functional theory (DFT) calculations to optimize the structure of the Cu/UiO-66-a model shown in Supplementary Fig. 11. The results are reported as Supplementary Information. The average Cu–Zr and Cu–O distances at the interface were calculated to be 3.28 and 2.03 Å, respectively. These values are in excellent agreement with the EXAFS fitting distances of 3.23 and 1.99 Å (Cu–Zr and Cu–O in Supplementary Table 2, respectively). The DFT results (Supplementary Data) also indicate that while the Cu atoms toward the center of the particle are almost neutral (only slightly negative or positive), the Cu atoms forming the Cu–O–Zr bonds have a significant formal positive charge and as such may be more Lewis acidic.

**Fig. 3:**
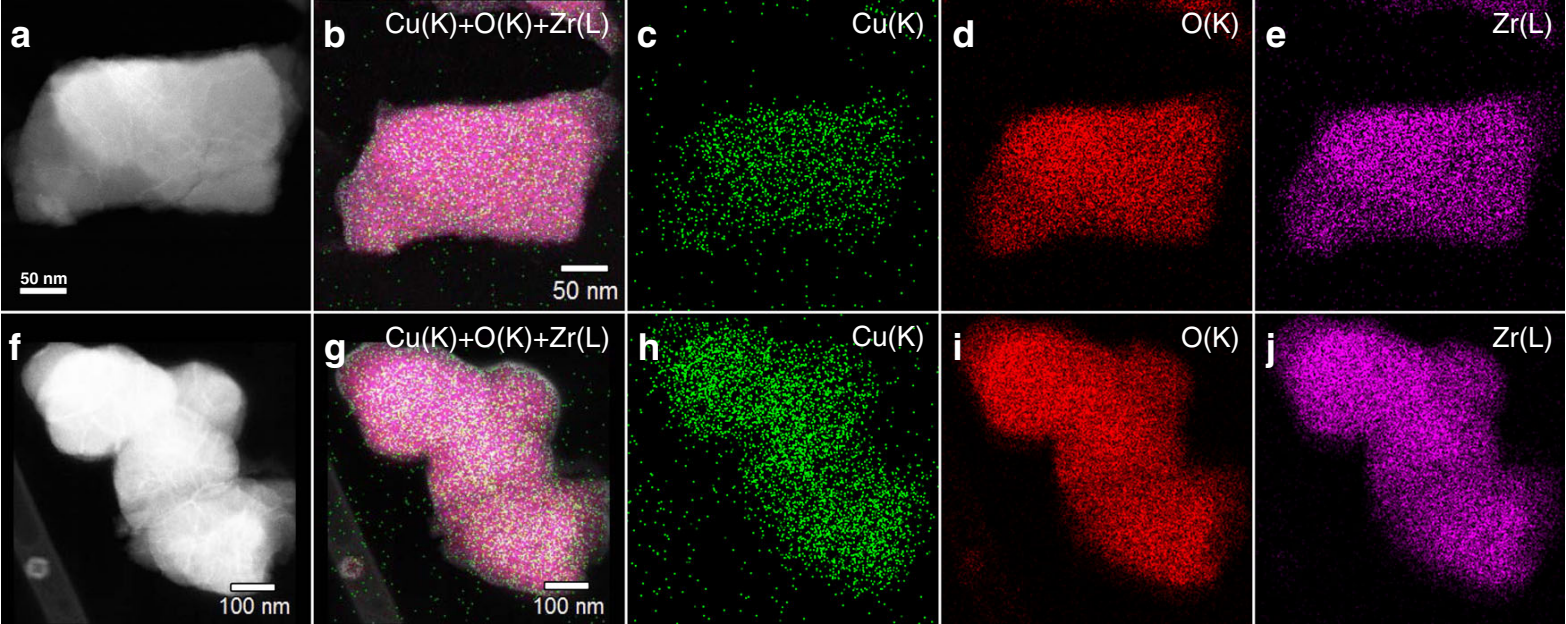
Microscopy characterization of Cu/UiO-66-a. HAADF-STEM-EDX images (**a**–**e**) of Cu/UiO-66-a showing the homogeneous distribution of Cu and Zr. The HAADF-STEM-EDX images (**f**–**j**) of Cu/UiO-66-a used at 250 °C show that the distributions of Cu and Zr within UiO-66 remain homogeneous after the reaction.

In agreement with the high dispersion of Cu deduced by the EXAFS fitting, we did not detect Cu nanoparticles sufficiently in size to be differentiated from the Zr background in electron microscopy analysis (Supplementary Fig. 12). High-angle annular dark field scanning transmission electron microscopy coupled with energy dispersive X-ray spectrometer (HAADF-STEM-EDX), on the other hand, showed that Cu is homogeneously distributed in the MOF (Fig. 3a–e). This homogenous distribution of Cu nanoparticles leaves the porous structure of the Cu-containing MOF as accessible as the parent MOF as shown by the isotherms of N_2_ physisorption (Supplementary Fig. 3).

EXAFS analysis of the environment and structure of Cu particles in Cu/UiO-66-b (Fig. 2b) showed that the average coordination number for the Cu–Cu_1_ path was 6.6 ± 0.7 (Supplementary Table 3), corresponding to a size of 26 ± 12 Cu atoms and a fraction of directly accessible Cu of 0.84^23^. Thus, we conclude that the particle size was nearly identical in both samples. In this case, however, the increase of *k*^*n*^ weighting did not lead to an increase in any of the intensities of the Fourier-transform (Supplementary Fig. 13), indicating a low fraction of Cu in the proximity of Zr. This agrees well with the fact that the XANES indicate that 90% of Cu is metallic in Cu/UiO-66-b and that a Cu–Zr path was not required to fit the EXAFS data (Supplementary Fig. 14 and Supplementary Table 1). We do not discard the presence of Cu–O–Zr bonds in this sample. The characterization, however, allows us to conclude that their abundance is too low to contribute to the X-ray absorption spectra.

We explored the impact of the Cu particle size by changing the concentration of ion-exchanged Cu. Materials with Cu concentrations of 0.04 wt.% and 7.6 wt.% are denoted Cu/UiO-66-a-low and Cu/UiO-66-a-high, respectively. The EXAFS and the Fourier-transformed spectra are shown in Fig. 2c–d. A Cu–Cu scattering path was not observed for Cu/UiO-66-a-low, indicating that Cu was present as a single atom site. Fitting shows that this Cu atom coordinates with 3**–**4 O atoms, of which two bind to Zr atoms of the Zr_6_O_8_ node (Supplementary Table 4). For Cu/UiO-66-a-high, the Cu–Cu paths show the presence of relatively large metallic Cu species. The Cu–Cu coordination number of 10 (Supplementary Table 5) indicates an average fraction of accessible Cu of 0.39 and a particle diameter larger than 2 nm^5,24^. Cu–O and Cu–Zr paths could not be fitted into the EXAFS spectra. Thus, although likely present, the concentration of Cu–O and Cu–Zr neighbors is small compared to concentration of Cu–Cu neighbors. We speculate that Cu particles vary statistically in size at such high-Cu contents. Thus, by varying the Cu loading, we synthesized catalysts with only Cu–O–Zr sites (Cu/UiO-66-a-low) and a catalyst with mainly metal-like Cu particles with a minor content of Cu–O–Zr sites (Cu/UiO-66-a-high).

### Catalytic activity and selectivity for hydrogenation of CO_2_

**Fig. 4:**
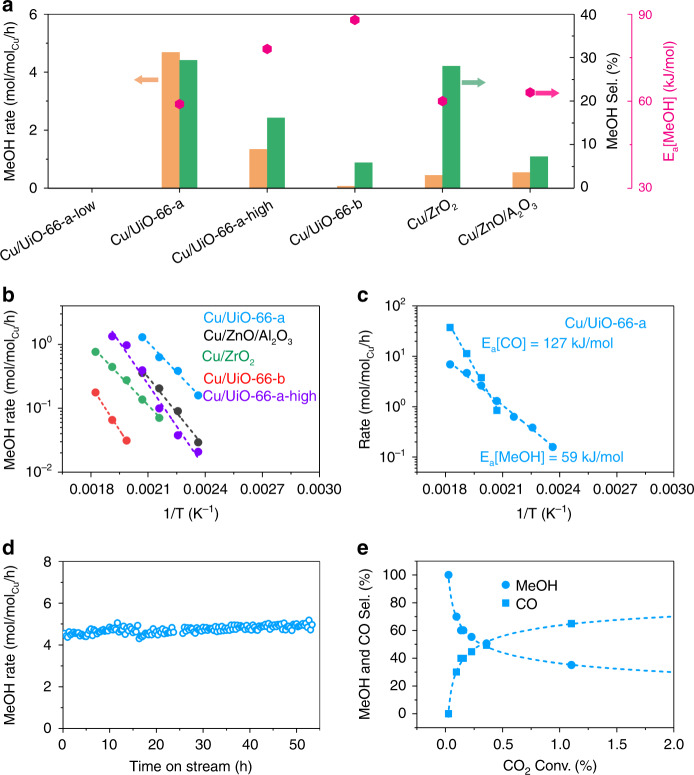
Catalytic performance of selected materials. **a** Rates of methanol (MeOH) production (orange bars), methanol selectivity (green bars) on selected catalysts at 250 °C and 32 bar, and activation energy (*E*_a_, pink points). **b** Arrhenius plots for methanol production on selected catalysts. The colors of the lines match the colors of the names of the materials. **c** Methanol and CO production on Cu/UiO-66-a. **d** Rate of methanol production on Cu/UiO-66-a as a function of time on stream for reaction conditions of 250 °C, 32 bar and CO_2_/H_2_/N_2_ = 7/21/1 mL/min. **e** The methanol and CO selectivity as functions of CO_2_ conversion for Cu/UiO-66-a at 190 °C and 32 bar. The CO_2_ conversion is varied by changing the gas space velocity.

Catalysis was studied under differential conditions (i.e., CO_2_ conversions below <5%). Methanol and CO were the only products observed on all catalysts under the explored temperatures and pressures (i.e., at concentrations far from equilibrium [Supplementary Fig. 15]). Cu/UiO-66-a showed a remarkably high rate of methanol production; that is, 4.7 mol_MeOH_/mol_Cu_/h at 250 °C and 32 bar, which is almost an order of magnitude higher than the rates with a reference Cu/ZrO_2_ (and with the benchmark Cu/ZnO/Al_2_O_3_) and almost two orders of magnitude higher than with Cu/UiO-66-b (Table 2). Upon normalizing the rates of methanol production to the concentration of exposed Cu atoms (Supplementary Table 6), the large differences in rates over Cu/UiO-66-a and Cu/UiO-66-b remained at 5.6 h^−1^ and 0.08 h^−1^, respectively. The normalized rate of methanol production on Cu/UiO-66-a was three times higher than the rates on the reference Cu/ZrO_2_ and the benchmark Cu/ZnO/Al_2_O_3_ catalysts (1.7 h^−1^ and 1.9 h^−1^, respectively). This difference is caused by specific anchoring of metallic Cu particles. The selectivity to methanol on Cu/UiO-66-a was 29% compared to 7% on the benchmark catalyst (Fig. 4a, b and Supplementary Table 7) at comparable conversion at 250 °C.

These variations in conversion rates and selectivity show that a material with sufficiently small metallic Cu particles and a large fraction of Cu–O–Zr interface sites (Cu-UiO-66-a) is an active and selective catalyst for methanol synthesis. Importantly, the formation rate of methanol on Cu/UiO-66-a was stable over 50 h on stream (Fig. 4d). Accordingly, the XANES spectrum of the spent catalyst was identical to the fresh catalyst (Supplementary Fig. 16), which indicates that the proportions of metallic and cationic Cu do not change during the reaction. The microscopy characterization of the spent catalyst showed that the even Cu distribution was retained during the time on stream (Fig. 3f–j). These observations indicate that Cu particles and their binding with the ZrO_2_ nodes are highly stable. The shape of the N_2_ physisorption isotherms and the X-ray diffraction patterns of the used catalyst were identical to the fresh material (Supplementary Figs. 3 and 4). Thus, the MOF is structurally stable at the conditions explored in this work.

Cu/UiO-66-a-high and Cu/UiO-66-b were 1 to 2 orders of magnitude less active than Cu/UiO-66-a. The selectivity to methanol on Cu/UiO-66-a was 30% at 250 °C but only 18 and 8% over Cu/UiO-66-a-high and Cu/UiO-66-b, respectively. Thus, the formation of methanol is directly related to the concentration of interface sites between Cu and ZrO_2_. However, Cu/UiO-66-a-low, with 100% (but cationic) Cu binding to Zr_6_ nodes, showed only negligible rates of methanol formation, indicating that the interface sites alone are insufficient to reduce CO_2_ to methanol. It is worth noting that this catalyst with only single Cu atoms was, however, active and selective for the reverse water-gas shift reaction.

As shown in Fig. 4c, CO formation is favored at high temperature, while methanol formation is favored at relatively low temperature. The comparison of conversion and yield on Cu/UiO-66 shows that initially only methanol was formed, and that the generation of CO seems a secondary reaction of methanol (Fig. 4e). We note in passing that Cu/ZnO/Al_2_O_3_ shows a similar tendency as Cu/UiO-66 for this secondary reaction (Supplementary Fig. 17). This suggested that, in contrast to earlier reports^25^, selectivity may not be limited by a parallel route to water-gas-shift. Instead, CO could form under the present reaction conditions via methanol dehydrogenation and formaldehyde decomposition. To verify this hypothesis, we converted methanol over selected catalysts. We observed that CO was indeed formed at comparable rates that observed during the hydrogenation of CO_2_ (Supplementary Table 8), which confirmed that methanol dehydrogenates to CO under conditions of CO_2_ hydrogenation. As the apparent activation for CO formation was 127 kJ/mol for the catalyst having the largest fraction interface sites and 147 kJ/mol when using methanol as feed, a fraction of the CO formed could also result from reverse water-gas-shift, having typically a low apparent activation energy.

Cu/UiO-66-a, Cu/ZrO_2_, and Cu/ZnO/Al_2_O_3_ showed the same activation energy of ~60 kJ/mol for methanol synthesis (Table 2 and Fig. 4a). Thus, we hypothesize that the mechanism and the rate-determining steps are identical for these catalysts, which suggests that the concentration of active sites determines the methanol formation rates. In contrast, Cu/UiO-66-b showed an activation energy of 88 kJ/mol for methanol synthesis, which is consistent with the lower activity of a pure Cu phase. The activation energies for CO formation varied from 58 kJ/mol to 127 kJ/mol for the catalysts listed in Table 2, indicating that the secondary reduction had to overcome a higher activation barrier, not untypical for the endothermic step of methanol dehydrogenation. Three other potential catalysts—(1) parent UiO-66, (2) a physical mixture of Cu clusters and UiO-66, and (3) zeolites (MOR and SSZ-13 with varying Si:Al ratios) exchanged with Cu—were either completely inactive or produced only CO (Table 2 and Supplementary Table 7).

**Table 2:** Catalytic performance of selected tested materials.

Catalysts^a^	MeOH rate [mol/mol_Cu_/h]^b^	CO rate [mol/mol_Cu_/h]^b^	MeOH/CO [mol/mol]^b^	*E*_a_[MeOH] [kJ/mol]		*E*_a_[CO] [kJ/mol]
Cu/UiO-66-a	4.68	11.24	0.42	59 ± 2	127 ± 6	
Cu/UiO-66-b	0.07	1.12	0.06	88 ± 4	113 ± 9	
Cu/ZrO_2_	0.44	1.13	0.39	60 ± 2	69 ± 8	
Cu/UiO-66-a-low^c^	0.01	20.62	0.0006	-	113 ± 10	
Cu/UiO-66-a-high	1.34	6.94	0.19	78 ± 6	105 ± 5	
Cu Nanoparticles on UiO-66^d^	0	1.06	0	-	-	
Cu/MOR	0	0.26	0	-	101 ± 11	
Cu/SSZ13-1 (Si/Al = 36)	n.d.	5.81	0	-	67 ± 2	
Cu/SSZ13-2 (Si/Al = 24)	0.07	11.31	0.01	-	66 ± 1	
Cu/SSZ13-3 (Si/Al = 6)	n.d.	0.95	0	-	58 ± 8	
Cu/ZnO/Al_2_O_3_	0.54	6.90	0.08	63 ± 6	114 ± 2	

^a^Conditions: 250 °C, 32 bar, CO_2_/H_2_/N_2_ = 7/21/1 mL/min.

^b^Rates were calculated based on the Cu concentrations of the catalysts.

^c^The catalytic test was performed with the same amount of Cu as for Cu/UiO-66-a by increasing the catalyst mass.

^d^This was a physical mixture of Cu NPs and UiO-66.

We compared the rates that we observed in this work over the MOF-based catalysts and reference materials with rates reported in open literature for a variety of Cu-containing catalysts in Supplementary Table 9^3,26–36^. The diversity of reaction conditions does not allow for a direct comparison. However, on the Cu content basis, the high dispersion of Cu particles interacting with ZrO_2_ nodes in Cu/UiO-66-a allows the rates of methanol production from CO_2_ to be among the highest rates reported at similar conditions.

### Adsorption of CO_2_ as critical property

**Fig. 5:**
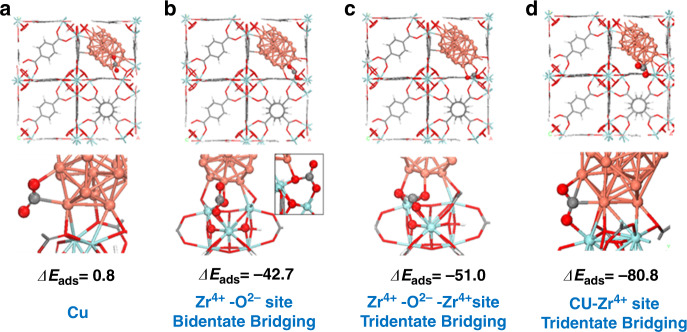
Adsorption energies (Δ*E*_ads_ in kJ/mol) of CO_2_ at the Cu/UiO-66-a interface. **a** Cu only sites, **b** Zr^4+^-O^2-^ sites on the Zr_6_O_8_ nodes in a bidentate bridging mode, **c** Zr^4+^-O^2-^- Zr^4+^ sites on the Zr_6_O_8_ nodes in a tridentate bridging mode, and **d** Cu-Zr^4+^ interfacial sites.

The adsorption and retention of CO_2_ is a critical property, because for a given site concentration, the sorption of CO_2_ on the surface under reaction conditions will influence the concentration term in the kinetics. To understand this, we carried out DFT calculations to examine the adsorption of CO_2_ on (1) Cu sites on the Cu cluster, (2) Zr^4+^-O^2-^ sites of the Zr_6_O_8_ nodes as a bidentate complex, (3) Zr^4+^-O^2-^-Zr^4+^ sites of the Zr_6_O_8_ nodes as a tridentate complex, and (4) Cu/ZrO_2_ interfacial Cu-Zr^4+^ sites (Fig. 5). The binding of CO_2_ is not energetically favored on the Cu metal particle (Fig. 5a), as the adsorption energy (Δ*E*_ads_) is slightly positive. This is consistent with the weak adsorption of CO_2_ found experimentally on Cu surfaces^27^. The adsorption of CO_2_ to the O^2−^ or Zr^4+^-O^2-^ sites of the Zr_6_O_8_ node to form a bidentate (Fig. 5b) or tridentate (Fig. 5c) carbonate is considerably stronger, resulting in adsorption energies of –42.7 and –51.0 kJ/mol, respectively, consistent with experimental results^37,38^. The adsorption of CO_2_ at the Cu-Zr^4+^ interfacial sites (Fig. 5d) is significantly stronger (Δ*E*_ads_ = −80.8 kJ/mol) than the adsorption on the Cu nanoparticles and ZrO_2_ nodes. CO_2_ is stabilized via electron transfer from the Cu nanoparticle to CO_2_ upon the binding of C to the metal along with the stabilization of the negatively charged oxygens by the interfacial Lewis acid Zr^4+^ and Cu sites.

Experimentally, we measured the heat of adsorption of CO_2_ by calorimetry on Cu/UiO-66-a to be 92 ± 20 kJ/mol, which is in excellent agreement with the theoretical value of Δ*E*_ads_ for strongly adsorbed species (i.e., −80.8 kJ/mol). This shows that the theoretical calculations capture the interactions of CO_2_ with the catalyst. We could not quantify the heat of adsorption of CO_2_ on Cu/UiO-66-b or the parent UiO-66 experimentally because the heat signal was below detection limits, which indicated weak interactions with CO_2_.

In temperature-programmed desorption experiments, we observed the desorption of CO_2_ (Supplementary Fig. 18) from the parent MOF occurring with a maximum at ~70 °C. In addition to this low-temperature desorption, the profiles of Cu/UiO-66-a and Cu/UiO-66-b showed CO_2_ desorption signals at high temperature. For Cu/UiO-66-a, we observed CO_2_ desorption extending from 200 °C until the end of the experiment with a shoulder at ~255 °C and a main peak at ~300 °C. For Cu/UiO-66-b, we observed weak CO_2_ desorption signals from ~250 to 350 °C. By comparing experiments with DFT calculations, we attribute the CO_2_ peak observed at low temperature to CO_2_ desorbing from ZrO_2_ nodes. The signals at high temperature are attributed to CO_2_ desorbing from Cu-O^2-^-Zr^4+^ sites. We attribute the wide temperature range for CO_2_ desorption to a distribution of carbonate and bicarbonate species (observed by in situ IR spectroscopy as shown below), which were not considered in our calculations.

The combination of DFT and experimental desorption results clearly showed that Cu ions at the interface and in particular the Cu-Zr^4+^ sites lead to the strong interactions. The CO_2_ binds to the interfacial Cu-Zr^4+^ sites that coordinate to the carbon and oxygen atoms, respectively allowing for partial electron transfer from the Cu into the CO_2_, which is stabilized by the coordination of the negatively charged oxygens and the Zr^4+^ cations. This is consistent with previous studies that show the strong binding of O_2_ at interfacial Au-Ti^4+^ sites, where Au transfers electron density into the antibonding states of O_2_ while Ti^4+^ concertedly adds to the oxygen to stabilizes the negative charge that forms on the oxygens^39^. A similar CO_2_ adsorption state was calculated to be most stable at the Cu/ZrO_2_ interface^3^. The amount of CO_2_ desorbing from Cu/UiO-66-a above 200 °C was 74 μmol per gram or 0.33 mol_CO2_/mol_Cu_. Thus, Cu/UiO-66-a adsorbs at least 0.46 molecules of CO_2_ per Cu atom in the sample, which equals ∼0.58 molecules of CO_2_ per surface Cu atom or 1.53 molecules of CO_2_ per Cu atom at the interface with ZrO_2_ nodes. However, due to the lack of the interface sites, Cu/UiO-66-b adsorbed only 0.04 molecules of CO_2_ per Cu atom in the sample (i.e., two orders of magnitude less than Cu/UiO-66-a, which is in good agreement with the difference in reactivity of the two materials).

We addressed the reaction mechanism by monitoring the products desorbing from UiO-66, Cu/UiO-66-a and Cu/UiO-66-b in H_2_ (after adsorbing CO_2_ at room temperature) and by in situ IR spectroscopy. The desorption experiments showed that only CO_2_ desorbed from the parent UiO-66 (Supplementary Fig. 18). The desorption from Cu/UiO-66-a showed CO_2_ (*m*/*z* = 44), methanol (*m*/*z* = 31, 0.13 mol_methanol_/mol_Cu_ in Cu/UiO-66-a), and small amounts of fragments that were assigned to HCOOH (*m*/*z* = 46) and HCHO (*m*/*z* = 30) (Supplementary Fig. 18 and Supplementary Table 10) appearing at ~290 °C. In contrast, the desorption from Cu/UiO-66-b showed only CO_2_ (*m*/*z* = 44) and HCOOH (*m*/*z* = 46). These observations allow us to conclude that the bound CO_2_ subsequently undergoes stepwise hydrogenation with the linked metallic Cu particles being the sites that are able to dissociate H_2_ and provide hydrogen atoms. The observation of HCOOH and HCHO during temperature-programmed desorption and reaction of CO_2_ indicates a path involving stepwise hydrogenation to formate, acetal, and methoxy species in agreement with a general pathway such as the one proposed in Supplementary Fig. 19^3,10,40^. At high temperatures, methanol is reversibly dehydrogenated and the resulting formate decomposes to H_2_ and CO^41^.

**Fig. 6:**
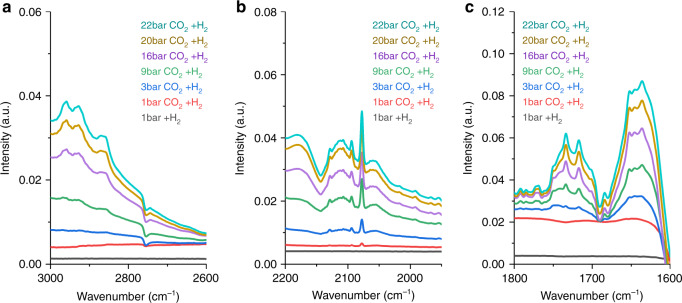
In situ IR spectra of Cu/UiO-66-a exposed to CO_2_ and H_2_. We used a CO_2_/H_2_ mol ratio of 1:3 from 1 to 22 bar at 200 °C. **a** Range of 3200–2600 cm^−1^ showing the formation of methoxy groups; **b** range of 2200–1950 cm^−1^ showing CO species adsorbed on various types of Cu sites; **c** and range of 1800–1600 cm^−1^ showing the formation of bicarbonate/carbonate species. The colors of the lines match the color of the conditions described in the insets.

The in situ IR spectra of Cu/UiO-66-a (Fig. 6) exposed to CO_2_ and H_2_ at high pressure (1–22 bar) showed the formation of gas-phase CO and CO adsorbed on different Cu sites. The bands around 2180 and 2120 cm^−1^ correspond to gas-phase CO. The bands at 2093, 2078, and 2060 cm^−1^ are linearly adsorbed CO molecules on metallic Cu species^42,43^. The band at 2129 cm^−1^ is attributed to CO adsorbed on cationic Cu^42,43^. The spectra show the variety of adsorption sites available in the catalyst and the cationic character of Cu in some of those sites as calculated by theory. We also observed bicarbonate/carbonates (1750–1690 cm^−1^ and 1680–1600 cm^−1^)^44^ and methoxy species (i.e., two pairs of bands at 2960–2930 cm^−1^ and 2865 cm^−1^, corresponding to the ν(CH_3_) and δ_s_(CH_3_) vibrations)^45^. Thus, we observed the products of the reaction on the best performing catalyst, but we could not identify the intermediates via these experiments. A detailed study of this reaction pathway goes beyond the purpose of the current contribution and a detailed mechanistic study is currently underway.

## Discussion

We show that direct bonding between ZrO_2_ and Cu particles is mandatory for the catalytic reduction of CO_2_ to methanol with Cu-UiO-66 catalysts. Thus, the nanoscopic Zr_6_O_*x*_ represents an upper limit to generate a maximum concentration of such an interface.

We conclude that constituents of the Zr–O–Cu interface are at least part of the active site that strongly adsorbs CO_2_. The high concentration of chemisorbed CO_2_ (which is not met when Cu and Zr_6_O_8_ are only in close proximity) and the sites for reduction at the interface are hypothesized to be the cause for the much higher activity of catalysts with a high concentration of Zr–O–Cu interface sites. In the absence of the hydrogenating function of Cu clusters, Zr–O–Cu sites (single Cu^δ+^ sites) maintain the high-CO_2_ adsorption capacity but show only reduction of CO_2_ to CO. For a series of Cu-based catalysts, the apparent energies of activation for methanol production are constant despite very different rates, demonstrating that the catalytic activity is related to the concentration of strongly interacting active sites complemented by an equilibrated H_2_ activation. The much higher and varying activation energies for CO formation point to different pathways on the tested catalysts, including the reverse water-gas-shift reaction and decomposition of methanol.

The highly dispersed Cu particles in MOFs are synthesized by ion exchange of Cu^2+^ cations at OH groups of under-coordinated Zr^4+^ of the ZrO_2_ nodes. The chemical bonds formed between Cu^2+^ and the MOF are preserved to a significant extent upon reduction, eventually leading to sub-nanometer Cu particles with a high proportion of surface Cu atoms covalently binding the nodes. The synthesis strategy opens a controlled pathway for chemically grafted Cu particles and likely of other metals that retain non-reduced metal cations at the interface and a fully reduced metal particle for the fraction that is not in direct chemical contact with the oxide support.

## Methods

### Chemicals

All chemicals were purchased from Sigma Aldrich and used without further treatment. Deionized water was produced by a Milli-Q lab water system.

### Catalyst synthesis

Samples of the metal organic framework (MOF) UiO-66 with controlled defect concentrations were prepared according to refs. ^16–18^. Calibrated N_2_-physisorption, used to estimate the defect concentration, indicates that approximately 1 of the 12 carboxylate linkers is missing in the MOF (equivalent to 0.5 missing linkers per unit cell)^16–19^. Missing linkers lead to the formation of acetate and/or −OH/ − OH_2_ groups at the Zr_6_ node of UiO-66^16^. The –OH and −OH_2_ groups are prone to react with metal ions via cation exchange (Supplementary Fig. 1a).

Details of the UiO-66 synthesis are as follows. We used a solvo-thermal procedure at 80 °C. We dissolved 8.39 g ZrCl_4_ (36 mmol) in 500 mL *N,N*-dimethylformamide (DMF) by rigorous stirring in a 2 L Parr autoclave. Then 66.6 mL 37 wt.% HCl aqueous solution was added to the reactor until the mixture became clear. In a separate 1 L glass bottle, 8.31 g terephthalic acid (50 mmol) was dissolved in 500 mL DMF by stirring. The solution of terephthalic acid was then added slowly to the Parr autoclave. The autoclave was then sealed and kept at 80 °C for 18 h. The resulting product was separated and washed with DMF three times and with acetone five times. After synthesis, the UiO-66 product was dried in a vacuum oven at 80 °C for 12 h. The yield of UiO-66 was about 86%.

The Cu/UiO-66-a material was prepared by ion exchange with a solution of Cu acetate for which Cu(OH)^+^ at pH 5^14^ was in contact with the OH groups on the Zr_6_ nodes (Supplementary Fig. 1b). In a typical experiment, 0.5 g UiO-66 was mixed with 300 mL aqueous solution of copper acetate (0.01 M). The suspension was stirred for 24 h at room temperature. The pH of the solution was 5.0–6.0 during this ion exchange. After the exchange, the product was separated by centrifugation and washed three times with deionized water. Then, the product was suspended in 50 mL acetone for 12 h to exchange the solvent. This step was repeated three times. The solvent-exchanged sample was then collected and dried at 120 °C in a vacuum oven for 12 h. The resulting Cu content was 1.4 wt.% Cu (i.e., ~0.5 Cu atoms per Zr_6_ node [Supplementary Table 1]). This is consistent with the decrease in concentration of OH groups induced by the defects. Comparing the infrared (IR) spectra of the parent UiO-66 to that of Cu/UiO-66-a showed that the band at 3674 cm^−1^ decreased in intensity as Cu(OH)^+^ exchanged with protons of the Zr_6_ node OH groups (Supplementary Fig. 1). Two additional samples, in particular Cu/UiO-66-a-low and Cu/UiO-66-a-high, were synthesized using the same procedure in which the concentration of copper acetate in aqueous solution was adjusted to control Cu loadings.

The Cu/UiO-66-b and Cu/ZrO_2_ catalysts were synthesized by impregnation. The parent UiO-66 for Cu/UiO-66-b was the same as for Cu/UiO-66-a. We purchased ZrO_2_ nanopowder (<100 nm, monoclinic, 20–30 m^2^/g) from Sigma Aldrich. In a typical procedure, 0.5 g UiO-66 or ZrO_2_ was mixed with 10 mL aqueous solution of copper nitrate (0.015 M). The mixture was stirred and dried in a water bath at 80 °C. Then the material was vacuum dried at 100 °C for 12 h. The resulting Cu/ZrO_2_ precursor was calcined in air at 450 °C for 4 h. The resulting Cu/UiO-66-b was treated in helium flow at 250 °C for 4 h.

The copper/mordenite (Cu/MOR) catalyst was prepared by ion exchange of H-MOR with aqueous solution of copper acetate (0.01 M). The H-MOR was obtained by calcining commercial NH_4_-MOR zeolite in air at 500^o^C for 8 h. The procedure for the Cu/MOR is similar to that for Cu/UiO-66-a.

Na/SSZ-13 zeolites with Si/Al ratios of 6, 24, and 36 were prepared with a hydrothermal method. In a typical synthesis of Si/Al = 36 zeolite, the procedure is as follows. First, 0.8 g NaOH (Sigma Aldrich, ≥99%) was dissolved in 38 mL deionized water. Then 17.1 g of TMAda-OH (Sachem Inc., 25% *N,N,N*-trimethyl-1-adamantyl ammonium hydroxide) was added as the structure directing agent. Then 0.5 g Al(OH)_3_ (Sigma Aldrich, ~54% Al_2_O_3_) was added to the solution and stirred at 400 rpm until it was completely dissolved. Next, 40 g LUDOX HS-30 colloidal silica (Sigma Aldrich, 30 wt.% suspension in H_2_O) was added to the mixture slowly until a uniform white gel formed. The gel was transferred and sealed in a 125 mL Teflon-lined stainless-steel autoclave with a magnetic stirring bar inside. The autoclave was heated in a sand bath on a hot-plate stirrer. The mixture was continuously stirred at 160 °C at 400 rpm for 4 days. The precipitate was separated by centrifugation and washed three times with deionized water. The final products were dried at 60 °C overnight under N_2_ gas flow. This was followed by calcination at 650 °C for 5 h in air to burn the residue structure directing agent.

For Si/Al = 6 and Si/Al = 24 zeolites, the procedures were similar, but we modified the amounts of Al(OH)_3_ and HS-30 colloidal silica. For Si/Al = 24, the amount of added Al(OH)_3_ was increased to 0.75 g, while the amount of other chemicals was unchanged. For Si/Al = 6, the amount of added Al(OH)_3_ was further increased to 1.5 g; at the same time, HS-30 was decreased to 20 g. To balance the water loss, an additional 14 mL deionized water was added.

The Na/SSZ-13 zeolites were ion-exchanged to NH_4_/SSZ-13 form before they could be used for Cu ion exchange. The Na/SSZ-13 zeolites were mixed with 0.1 M NH_4_NO_3_ solution and heated at 80 °C for 2 h with continuous stirring. The ion-exchange process was repeated once to make sure the Na^+^ ions were fully removed. The obtained NH_4_/SSZ-13 was dried at 60 °C overnight under N_2_ gas flow.

The Cu/SSZ-13 catalysts were prepared by ion exchange between NH_4_/SSZ-13 and Cu(NO_3_)_2_ solutions. We added 0.2 g Cu(NO_3_)_2_·2.5H_2_O to 100 mL deionized water and mixed this with 3 g zeolite. The mixture was continuously stirred at 80 °C for 2 h. The water was then evaporated by heating the solution to 150 °C under stirring to obtain a dry powder. The as-prepared Cu/SSZ-13 catalysts were calcined at 550 °C for 5 h.

The benchmark methanol synthesis Cu/ZnO/Al_2_O_3_ catalyst (F51-8PPT) was purchased from Synetix (now Johnson Matthey).

Cu nanoparticles were incorporated into UiO-66 by a two-step procedure involving (1) synthesizing the Cu nanoparticles (NP) and (2) mixing the Cu NPs with UiO-66. The Cu NPs were prepared by a method adopted from the literature^46^. In a typical procedure, 73 mg copper nitrate was added to the container with 20 mL 0.25 M NaOH/ethylene glycol solution. The mixture was rigorously stirred under N_2_ flow at 160 °C. The resulting brown precipitate was separated and washed eight times with acetone and water. To mix Cu NPs with UiO-66, 14 mg Cu NPs was ultrasonically dispersed in 20 mL acetone. Then 986 mg UiO-66 was added to the suspension of Cu NPs. The resulting mixture was stirred at room temperature until it was dry.

### Catalytic test

The catalytic test for CO_2_ hydrogenation was performed using a fixed-bed flow reactor, equipped with an online gas chromatography instrument (Agilent 7890B). In a typical procedure, 36 mg of catalyst material was loaded into the stainless-steel reactor. Prior to the catalytic tests, the catalysts were activated in situ by 25 vol.% H_2_/N_2_ flow at 200 °C for 30 min at a ramping rate of 2^o^C/min. After reaching the target reaction temperature, the catalyst was contacted with a mixture of CO_2_/H_2_/N_2_ (7/21/1 mL/min). The pressure of the reactor was kept at 32 bar.

The model experiment for methanol dehydrogenation to CO was performed using a fixed-bed flow reactor and by bubbling N_2_ flow (20 mL/min) through methanol at 20 °C (the saturated vapor pressure is 0.13 bar). The effluents were analyzed using an online gas chromatography instrument. The Cu/UiO-66-a, Cu/ZrO_2_, and UiO-66 were pretreated by the same procedure as used for the catalyst test for CO_2_ hydrogenation.

### Materials characterization

The elemental composition of the catalysts was determined by inductively coupled plasma atomic emission spectroscopy (Perkin Elmer 7300DV). Prior to the tests, the samples were digested in a mixture of HNO_3_/HCl/HF/H_2_O.

The X-ray absorption spectroscopy (XAS) experiment of Cu/UiO-66-a was carried out at the Pacific Northwest Consortium/X-Ray Science Division bending-magnet beamline at Sector 20 of the Advanced Photon Source at Argonne National Laboratory. All experiments were carried out in transmission mode with a focused beam (0.7 × 0.6 mm) delivering 10^10^ photons through the sample. A harmonic rejection mirror was used to reduce the effects of harmonics. A Cu foil was placed downstream of the sample cell as a reference to calibrate the photon energy of each spectrum. In a typical procedure, the catalyst was activated in hydrogen at 200 °C and loaded into a thin-walled glass capillary (0.02 mm) in an Ar-filled glove box. The capillary end was sealed with a propane flame.

The XAS experiments of other materials were carried out at the Stanford Synchrotron Radiation Lightsource. The Cu/UiO-66-b and Cu/UiO-66-a-high were measured in transmission mode while the Cu/UiO-66-a-low was measured in fluorescence mode.

The XAS data were processed using ATHENA, as part of the XAFS software package, to remove the background from the χ(*k*) oscillations. The Fourier-transform of the *k*-space extended X-ray absorption fine structure (EXAFS) data were fit to X-ray determined structure or theoretical models (FEFF9) using Artemis. The fits to the Cu K-edge EXAFS χ(*k*) data were weighted by *k*^2^ and windowed between 2.0 Å^−1^ < *k* < 12.0 Å^−1^ using a Hanning window with *dk* = 1.0 Å^−1^.

The microstructural observations of the activated and used catalysts were performed using an FEI Titan 80 − 300 transmission electron microscope (TEM) operated at 300 kV in the conventional TEM mode. The images were recorded using Gatan UltraScan1000 (2k × 2k). The Energy Dispersive Spectroscopy mapping analysis was performed with a probe corrected JEOL-ARM 200 F operated at 200 kV. The instrument is equipped with Centurio high-collection angle silicon drift detector (100 mm^2^).

The infrared spectra of the UiO-66 and Cu/UiO-66-a samples were recorded on a ThermoScientific Nicolete Fourier-transform infrared spectrometer equipped with CaF_2_ windows and a MCT detector with a resolution of 4 cm^–1^. The samples for IR measurements were prepared as self-supporting wafers with a density of approximately 5 mg/cm^2^. Upon loading in the IR cell, the samples were evacuated to 1.0 × 10^–7^ mbar at 130 °C for 3 h to remove physisorbed water. The background spectra were collected with an empty cell. The spectra were collected at 130 °C after evacuation and averaged over 128 scans.

### Adsorption–desorption studies

Adsorption of CO_2_ on Cu/UiO-66-a and UiO-66 followed by temperature-programmed desorption was performed in a flow reactor equipped with a mass spectrometer. The samples were first activated in H_2_ at 200 °C for 30 min and then purged with He until the baseline was stable. Then the samples were exposed to 8 bar CO_2_ for adsorption. After purging with H_2_ at 25 °C, the temperature was increased to 350 °C in flowing H_2_. Blank experiments (i.e., temperature treatment without prior exposure to CO_2_) were performed to exclude any potential impact of the desorption of fragments from the MOF. The signals measured in the blank experiments were subtracted from the profiles recorded after CO_2_ adsorption. The masses *m*/*z* = 44, 46, 30, and 31 were selected as representative of CO_2_, HCOOH, HCHO, and CH_3_OH, respectively. Those fragments are the most intense for such compounds and barely overlap among each other (Supplementary Table 10).

### Calorimetric experiments

The heat of CO_2_ adsorption was measured with a balance (SETARAM SENSYS EVO TG-DSC thermoanalyzer) equipped with a dosing and vacuum system. The sample was first loaded into the Al_2_O_3_ crucible of the balance and activated in H_2_ at 200 °C. Subsequently, the sample was degassed at 200 °C under 10^−7^ mbar for 30 min and then cooled to room temperature in vacuum. CO_2_ was dosed into the system for adsorption at 30 °C. The increase in weight and the heat flux were recorded during the adsorption. The heat of CO_2_ adsorption was obtained by dividing the heat by the mole of adsorbed CO_2_.

### In situ IR studies

In situ infrared spectra of Cu/UiO-66-a were measured in a ThermoScientific Nicolete Fourier-transform infrared spectrometer equipped with a high-pressure cell. A mass of 2 mg Cu/UiO-66-a was mixed with 8 mg SiO_2_ to achieve an optimum transmission in the range of 1750–1600 cm^−1^, and the mixture was pressed into a self-supported wafer. The cell was loaded with the wafer and flushed with H_2_ at a 20 mL/min rate at 1 bar and ramping up to 200 °C. A blank spectra was recorded at 200 °C in H_2_. Then CO_2_ (6.7 mL/min) and H_2_ (20 mL/min) with the CO_2_/H_2_ ratio of 1/3 were co-fed into the cell at 200 °C, and the spectra were recorded accordingly. The cell was then pressurized with the mixture of CO_2_ and H_2_ in the range of 1–22 bar, and the spectra were recorded.

### Calculation of defects and OH groups

The relationship between textural properties, derived from N_2_ adsorption, and the linker defects was reported in references^18,19^. A perfect UiO-66 has a surface area of 954 m^2^/g and a pore volume of 0.426 cm^3^/g. Every Zr_6_ node coordinates 12 carboxylate linkers. If 1 out of 12 linkers is removed from the ZrO_2_ cluster, the defective UiO-66 structure will have the surface area of 1433 m^2^/g and pore volume of 0.502 cm^3^/g. In the structure of UiO-66, the ditopic linker coordinates two Zr_6_ nodes by four Zr–O bridging bonds. Therefore, 1.1 missing linker will expose approximately 2.2 OH groups on a Zr_6_ node.

### Atomicity of Cu nanoparticles

The calculation of the atomicity of Cu clusters was based on the coordination numbers obtained from EXAFS fitting^23^. The first coordination shell does not vary significantly with the shapes of the particles (spherical, cubic, distorted cubic, and slab shapes). Equation 1 was derived from the correlation between the coordination number of the first metal–metal shell and the atomicity of metal particles:

1 $${\mathrm{CN}} = \frac{{8.981N_{{\mathrm{at}}}}}{{9.64 + N_{{\mathrm{at}}}}} + \frac{{3.026N_{{\mathrm{at}}}}}{{1462.61 + N_{at}}}$$

where CN is the coordination number of the first Cu–Cu shell, and *N*_at_ is the mean atomicity of the Cu atoms in the Cu clusters.

### Cu dispersion

The correlation between coordination number of the first Cu–Cu shell and Cu dispersion was derived from published data based on the spherical and raft-like shapes of the metal particles^5,24^.

### N_2_O titration

The N_2_O titration to determine the dispersion of metallic Cu was performed based reactions (i), (ii) and (iii) on a Micromeritics 2920 chemisorption instrument:

i $${\mathrm{CuO}} + {\mathrm{H}}_2 \to {\mathrm{Cu}} + {\mathrm{H}}_2{\mathrm{O}}$$

ii $$2{\mathrm{Cu}}_{({\mathrm{surface}})} + {\mathrm{N}}_2{\mathrm{O}} \to {\mathrm{Cu}}_2{\mathrm{O}}_{({\mathrm{surface}})} + {\mathrm{N}}_2$$

iii $${\mathrm{Cu}}_2{\mathrm{O}}_{({\mathrm{surface}})} + {\mathrm{H}}_2 \to 2{\mathrm{Cu}} + {\mathrm{H}}_2{\mathrm{O}}$$

In a typical procedure, the sample was loaded into the quartz tube and pretreated at 105 °C in He flow to remove adsorbates from the surface. The sample was reduced at 300 °C in a 10 vol.% H_2_/Ar (30 mL/min) flow for 60 min with a 5 °C/min rate. Then, it was exposed to 0.5 vol.% N_2_O/Ar flow for 60 min at 25 °C to oxidize the surface Cu to Cu_2_O. Finally, the sample was flushed with Ar before the second temperature-programmed reduction. Copper dispersion was calculated by dividing the number of titrated sites by the total number of copper atoms.

### EXAFS spectra simulation

EXAFS spectra of density-functional-optimized Cu models were simulated using ab initio scattering theory by applying approximate global disorder parameters (*σ*^2^), corresponding to 300 K. The computed coordinates were used to generate the primary input for the ab initio EXAFS scattering code (FEFF9) that includes all the single and multiple scattering paths out to 6 Å, resulting in several hundred scattering paths for each Cu atom in the structure^22^. An approximate treatment of the bond disorder at 300 K is applied by setting a universal value of the Debye–Waller factor, *σ*^2^ = 0.0035 Å^2^. The obtained spectra for each Cu atom in the cluster are then averaged, and an overall E_0_ is applied to match experimental values (oscillations in *χ*(*k*) converge at *k* = 0). While the global Debye–Waller factor is a good estimate of the first shell disorder, it is an overestimation of the order in the higher shells which manifests as an over-prediction of these amplitudes, although the atom positions predicted by the theory are correctly represented.

### Density functional theory calculations

Periodic density functional theory calculations were carried out using the CP2K code^47^. The PBE functional^48^ with D3 dispersion corrections^49^ were used to calculate the exchange–correlation energy. The DZVP-MOLOPT basis set was used with Goedecker et al. pseudopotentials^50^ with a plane wave cutoff energy of 400 Ry. The structures are optimized with fixed lattice constants. The CM5 charges are calculated using the Chargemol program^51^.

## Supplementary information

Supplementary Information

Description of Additional Supplementary Files

Supplementary Data 1

## Data Availability

The data that support the findings of this study are available from the corresponding author upon request.
