# Supplementary material for: Copper-zirconia interfaces in UiO-66 enable selective catalytic hydrogenation of CO2 to methanol
Source: Nat Commun. 2020 Nov 18;11:5849. doi: 10.1038/s41467-020-19438-w (PMC7674450; doi:10.1038/s41467-020-19438-w)
Supplement: Supplementary file 1 — Supplementary Information [file 41467_2020_19438_MOESM1_ESM.pdf]

Supporting Information

Copper-zirconia interfaces in MOF UiO-66 enable selective catalytic hydrogenation of CO_2 to methanol

Yifeng Zhu,[†,#] Jian Zheng,[†,#] Jingyun Ye,[‡,+] Yanran Cui,[†] Katherine Koh,[†] Libor Kovarik,[§] Donald M. Camaioni,[†] John L. Fulton,[†] Donald G. Truhlar,[‡] Matthew Neurock,[‡] Christopher J. Cramer,[‡] Oliver Y. Gutiérrez,[†,]* and Johannes A. Lercher,[†,]*

[†] Institute for Integrated Catalysis, and Fundamental and Computational Science Directorate, Pacific Northwest National Laboratory, Richland, Washington 99354, USA

[‡] Department of Chemistry, Minnesota Supercomputing Institute, and Chemical Theory Center, University of Minnesota, Minneapolis, Minnesota 55455, USA

[§] William R, Wiley Environmental Molecular Sciences Laboratory, Pacific Northwest National Laboratory, Richland, Washington 99354, USA.

[+] Present address: Department of Chemistry and Biomolecular Science, Clarkson University, Potsdam, New York 13699, USA.

johannes.lercher@pnnl.gov; oliver.gutierrez@pnnl.gov

[#] contributed equally.

Supplementary Note: XANES and EXAFS analysis

Oxidation states of Cu species

We used the position and intensity of the white line (8978 eV) in Cu K-edge X-ray absorption near edge structure (XANES) to characterize the environment and structure of Cu species. Cu/UiO-66-a exhibited a white line with intensity between those of Cu foil and copper oxides, indicating that Cu exists in a partially oxidized form. However, the typical pre-edge feature of Cu_2O was not observed, suggesting the absence of Cu^+ species. Therefore, we chose the Cu foil and CuO as the reference for the XANES linear combination fitting. The XANES data indicated that 66% metallic Cu was present in Cu/UiO-66-a (**Supplementary Figure 5** and **Supplementary Table 1**). In turn, the XANES results showed that approximately 34% of Cu atoms are in an apparently cationic state and may form a Cu-O-Zr bond with the nodes. For Cu/UiO-66-b (**Supplementary Figure 14** and **Supplementary Table 1**), a very similar XANES spectrum to that of Cu foil was observed; the corresponding linear combination fitting suggested that 91.2% Cu is metallic.

Cu-O-Zr bonding and location of Cu species

The comparison among Fourier-transformed imaginary $\chi(R)$ spectra of Cu/UiO-66-a with different k^n weightings allows differentiation of the low-Z and high-Z scattering elements around Cu, because scattering contributions from elements with higher mass increase as k weighting increases.[10, 16, 17] Zr is the only element present that is much heavier than Cu, which makes EXAFS feasible for discerning the contributions of Zr species out of Cu–Cu paths. As shown in **Supplementary Figure 6**, the spectra of Cu/UiO-66-a are compared with the spectra of the Cu foil reference at increased k weightings. A feature around 0.3 nm becomes more intense as the k weighting increases for Cu/UiO-66-a, while it is not present for Cu foil. The results suggest that this contribution stems from scattering by a nearby heavier element (i.e., Zr at the Zr-O-Cu interface).

The EXAFS spectra were fitted to determine the structural and chemical environment of Cu species. **Figure 2A** and **Supplementary Figure 7** show the results in both the amplitude and the imaginary parts. The prominent path at 0.22 nm in **Figure 2A** is attributed to the backscattering of the closest Cu–Cu coordination (denoted as $Cu–Cu_1$). The features at distances larger than 0.33 nm are attributed to the backscattering Cu–Cu paths of higher shells (denoted as $Cu–Cu_2$ and $Cu–Cu_{higher\ shells}$) that occur in Cu particles. Along the Cu–Cu backscattering paths, the features observed at ~0.15 nm and ~0.28 nm (not phase corrected) are attributed to the Cu–O and Cu–Zr paths, respectively (**Supplementary Figure 7**). The existence of a Cu–Zr path is further supported by comparing the fittings with Cu–Zr path and without Cu–Zr path for Cu/UiO-66-a (**Supplementary Figure 20**). The R-factor and Chi-square improved by about 50% (**Supplementary Table 1**) fitting plots are better in both imaginary and magnitude spaces (**Supplementary Figure 20**). The EXAFS fitting parameters are listed in **Supplementary Table 2**, and the corresponding EXAFS oscillations, which agree well with experiment, are shown in **Supplementary Figure 8**.

For Cu/UiO-66-b, however, the increase of k^n weighting did not lead to an obvious increase in any of the intensities of the Fourier transform (**Supplementary Figure 13**), indicating the absence of proximity of Cu and Zr. This is consistent with the XANES and EXAFS results that indicate that

more than 90% of Cu is metallic in Cu/UiO-66-b and that a Cu–Zr path was not required to fit the data (**Supplementary Figure 14** and **Supplementary Table 1**).

Atomicity and location of Cu species

It is well-established by theory and experiments that the coordination number of the metal–metal shells of metal particles is related to the atomicity or particle size.[8, 9] Assuming the subnanometer Cu clusters in the cage of UiO-66 are nearly spherical, the average coordination number derived from the first shell $Cu–Cu_1$ path is ~6.5 ±0.3, and this allowed us to determine that the particle contains 25 ±4 Cu atoms. This is equivalent to an average diameter of 0.7–0.8 nm and a fraction of exposed Cu of 0.85 (see **Supplementary Figures S9–S10** for the calculations and **Figure 2a** for the optimized geometry). These Cu clusters are likely located within the pores of UiO-66, as they are smaller than both the octahedral and defective pore sizes (~0.9 nm and 1.3–1.5 nm respectively). Filling the pores with Cu clusters is supported by the N_2 physisorption (**Supplementary Figure 3**). The calculated coordination numbers of Cu–O and Cu–Zr were 0.3–0.4, indicating that approximately 30% of the Cu atoms are bonded to the Zr_6O_8 nodes via Cu-O-Zr bridges (**Figure 2a**). In agreement with the high dispersion of Cu found in the EXAFS fitting, we did not find larger Cu nanoparticles in electron microscopy analysis (**Supplementary Figure 12**). High-angle annular dark field scanning transmission electron microscopy coupled with energy dispersive X-ray spectrometer, on the other hand, showed that Cu is homogeneously distributed across the MOF (**Figures 3a-e**).

EXAFS analysis of the environment and structure of Cu particles in Cu/UiO-66-b (**Figures 2b**) showed that the average coordination number for the $Cu-Cu_1$ path was 6.6 ±0.7 (**Supplementary Table 4**), corresponding to a size of 26 ±12 Cu atoms and a fraction of directly accessible Cu of 0.84.[8] Thus, we conclude that the particle size was nearly identical in both Cu/UiO-66-b and Cu/UiO-66-a samples.

Varying Cu atomicity

We explored the impact of the Cu particle size by changing the concentration of ion-exchanged Cu. Materials with Cu concentrations of 0.04 wt.% and 7.6 wt.% are denoted as Cu/UiO-66-a-low and Cu/UiO-66-a-high, respectively. The EXAFS and the Fourier-transformed spectra are shown in **Figures 2c-d**. A Cu–Cu scattering path was not observed for Cu/UiO-66-a-low, indicating monoatomic dispersion. The fitting further indicates that the Cu atom coordinates with three-to-four O atoms, of which two bind to Zr atoms of the Zr_6O_8 node (**Supplementary Table 5**). The XANES spectra of those isolated Cu atoms is close to the CuO standard, suggesting the cationic state of those isolated Cu atoms stabilized by Cu-O-Zr bonds (**Supplementary Table 1** and **Supplementary Figure 21**).

For Cu/UiO-66-a-high, the Cu–Cu paths show the presence of metal-like Cu particles. The Cu–Cu coordination number of 10 (**Supplementary Table 6**) indicates a fraction of accessible Cu of 0.39 and an average particle diameter larger than 2 nm.[15, 18] Cu–O and Cu–Zr paths could not be fitted into the EXAFS spectra. The XANES results also showed negligible contribution of Cu–O bonding, as all the Cu species are in a metallic state (**Supplementary Table 1** and **Supplementary Figure 22**). We conclude, therefore, that the concentration of Cu–O and Cu–Zr neighbors is small compared to the concentration of Cu–Cu neighbors. We hypothesize that these larger Cu particles

form in the pores and at external MOF surfaces (**Supplementary Figure 3**). Thus, by varying the Cu loading, we also synthesized catalysts with only Cu-O-Zr sites (Cu/UiO-66-a-low) and a catalyst with mainly metal-like Cu particles with a minor content of Cu-O-Zr sites (Cu/UiO-66-a-high).

Supplementary Table 1. Elemental composition and structure of catalysts.

Catalysts	Cu loadings (wt.%)	Zr/Cu (mol/mol)	Proportion of Cu^0 (%)[a]
Cu/UiO-66-a	1.40	12.2	65.7
Cu/UiO-66-a-low	0.04	431.3	30.9
Cu/UiO-66-a-high	7.62	2.1	100
Cu/UiO66-b	1.80	11.4	91.2
Cu/ZrO_2	1.43	—	—
Cu NPs on UiO-66	1.40	12.2	—
Cu/MOR	2.75	—	—
Cu/SSZ13-1 (Si:Al=36)	1.90	—	—
Cu/SSZ13-2 (Si:Al=24)	1.85	—	—
Cu/SSZ13-3 (Si:Al=6)	1.80	—	—
Cu/ZnO/Al_2O_3	41.20	—	—

[a] Proportion of metallic Cu (Cu^0) on the activated catalyst is derived from the linear combination fitting of the XANES results using Cu foil and CuO as references.

Supplementary Table 2. EXAFS fitting parameters of activated Cu/UiO-66-a.

Backscatterer	Coordination number	Distance (Å)	Debye-Waller factor ($\Delta\sigma^2$, Å^2)
Cu-O	0.3 (± 0.2)	1.99 (± 0.01)	0.006 (set)
Cu-Cu_1	6.5 (± 0.3)	2.56 (± 0.04)	0.010 (±0.001)
Cu–Zr	0.4 (± 0.2)	3.23 (± 0.02)	0.012 (±0.007)
Cu-Cu_2	3.2 (± 2.3)	3.62 (± 0.05)	0.010 (±0.007)

Note: Debye-Waller factors were fixed for Cu-O path during the EXAFS fitting to reduce the number of parameters. They were set to the corresponding values obtained from CuO standard.

Supplementary Table 3 EXAFS fitting results of activated Cu/UiO-66-b catalyst.

Backscatterer	Coordination number	Distance (Å)	Debye-Waller factor ($\Delta\sigma^2$, Å^2)
Cu-O	0.6 (± 0.3)	1.97 (± 0.03)	0.006 (set)
Cu-Cu_1	6.6 (± 0.8)	2.56 (± 0.008)	0.008 (± 0.001)
Cu–Zr	not detected	not detected	not detected
Cu-Cu_2	3.9 (± 1.1)	3.69 (± 0.05)	0.011 (± 0.010)

Note: Debye-Waller factors were fixed for Cu-O path during the EXAFS fitting to reduce the number of parameters. They were set equal to the corresponding values obtained from CuO standard.

Supplementary Table 4. EXAFS fitting results of activated Cu/UiO-66-a-low catalyst.

Backscatterer	Coordination number	Distance (Å)	Debye-Waller factor ($\Delta\sigma^2$, Å^2)
Cu-O	3.5 (± 0.3)	1.93 (± 0.03)	0.008 (± 0.001)
Cu-Cu_1	not detected	not detected	not detected
Cu–Zr	1.9 (± 0.8)	3.07 (± 0.17)	0.016 (± 0.005)
Cu-Cu_2	not detected	not detected	not detected

Supplementary Table 5. EXAFS fitting results of activated Cu/UiO-66-a-high catalyst.

Backscatterer	Coordination number	Distance (Å)	Debye-Waller factor ($\Delta\sigma^2$, Å^2)
Cu-O	not detected	not detected	not detected
Cu-Cu_1	10.0 (± 1.2)	2.58 (± 0.002)	0.010 (± 0.001)
Cu–Zr	not detected	not detected	not detected
Cu-Cu_2	5.1 (± 1.7)	3.61 (± 0.05)	0.013 (set)

Note: Debye-Waller factors were fixed for Cu-Cu_2 and Cu-Cu_3 paths during the EXAFS fitting to reduce the number of parameters. They were set to the corresponding values obtained from the Cu standard.

Supplementary Table 6. Dispersion of metallic Cu in selected catalysts and rates of methanol production normalized to the concentration of exposed Cu corresponding to those dispersion values.

Catalysts	Cu dispersion (%)[a]	MeOH production rates (h^{-1})
Cu/UiO-66-a	85	5.57
Cu/UiO-66-b	84	0.08
Cu/ZrO_2	26	1.69
Cu/UiO-66-a-low	100	0.01
Cu/UiO-66-a-high	39	3.44
Cu/ZnO/Al_2O_3	28	1.93

[a] The dispersion of Cu species for the MOF-based materials is calculated based on the EXAFS data. The Cu dispersion for Cu/ZrO_2 and Cu/ZnO/Al_2O_3 is determined by N_2O titration experiment.

Supplementary Table 7. Catalytic performance of tested materials.[a]

Catalysts	MeOH rate (mol/mol_{Cu}/h)[b]	CO rate (mol/mol_{Cu}/h)[b]	MeOH/CO (mol/mol)[b]	E_a[MeOH] (kJ/mol)	E_a[CO] (kJ/mol)
Cu/UiO-66-a	4.68	11.24	0.42	59±2	127±6
Cu/UiO-66-b	0.07	1.12	0.06	88±4	113±9
Cu/ZrO_2	0.44	1.13	0.39	60±2	69±8
Cu/UiO-66-a-low	not detected [e]	not detected [e]	-	-	-
Cu/UiO-66-a-low[c]	0.01	20.62	0.0006	-	113±10
Cu/UiO-66-a-high	1.34	6.94	0.19	78±6	105±5
Cu NPs on UiO-66[d]	not detected [e]	1.06	0	-	-
UiO-66	not detected	not detected	-	-	-
Cu/MOR	0	0.26	0	-	101±11
Cu/SSZ13-1 (Si:Al=36)	not detected	5.81	0	-	67±2
Cu/SSZ13-2 (Si:Al=24)	0.07	11.31	0.01	-	66±1
Cu/SSZ13-3 (Si:Al=6)	not detected	0.95	0	-	58±8
Cu/ZnO/Al_2O_3	0.54	6.90	0.08	63±6	114±2

[a] Conditions: 250 °C, 32 bar, $CO_2/H_2/N_2$ = 7/21/1 ml/min. [b] Rates were calculated based on the Cu concentrations of the catalysts. [c] The catalytic test was performed at a same amount of Cu as Cu/UiO-66-a by increasing the catalyst mass. [d] This is a physical mixture of Cu NPs and UiO-66. [e] Inactive for the reaction

Supplementary Table 8. Rates of CO formation when methanol and N_2 were used as feed (CO formation rate from MeOH) and rates of CO and methanol formation during the standard experiments of CO_2 hydrogenation (rates "from MeOH").

Catalysts	T (°C)	CO formation rate from MeOH (mol mol_{Cu}^{-1} h^{-1})	CO rate in CO_2 hydrogenation (mol mol_{Cu}^{-1} h^{-1})	MeOH rate in CO_2 hydrogenation (mol mol_{Cu}^{-1} h^{-1})
Cu/UiO-66-a	230	2.8	3.8	2.6
	250	9.8	11.2	4.7
	275	49.4	37.2	6.9
Cu/ZrO_2	230	0	0.8	0.3
	250	0	1.1	0.4
	275	1.1	2.7	0.8

Supplementary Table 9. Comparison of the activities of selected catalysts studied in this work with a commercial $Cu/ZnO/Al_2O_3$ catalyst and with materials based on Cu and ZrO_2 reported in literature. The columns labeled as T, P, H_2/CO_2, and GSHV are the reaction conditions. The columns labeled MeOH rate, MeOH sel., and E_a[MeOH] compile the results corresponding to MeOH production: rates, selectivity and activation energy.

Catalysts	Loading (wt. %)	T (°C)	P (bar)	H_2/CO_2 (mol/mol)	GSHV ($mL_{CO2}/g_{cat}/h$)	MeOH rate ($mol/mol_{Cu}/h$)	MeOH Sel. (%)[a]	E_a[MeOH] (kJ/mol)[a]
Cu/UiO-66-a (this work)	1.4	250	32	3	11667	4.68	29.6	59±2
Cu/ZrO_2 (this work)	1.4	250	32	3	11667	0.44	28.1	60±2
Cu/ZrO_2[19]	5	220	1	3	40000	1.71	28.3	n.d.[a]
Cu/ZrO_2[20]	32.9	240	20	3	6818	1.33	n.d.	n.d.
Cu/a-ZrO_2[21]	10.0	260	30	3	1800	1.74	42	23.3
Cu/t-ZrO_2[21]	10.0	260	30	3	1800	1.29	53	36.4
Cu/m-ZrO_2[21]	10.0	260	30	3	1800	0.36	48	63.5
Cu/ZrO_2[22]	2.3	230	25	3	4800	1.95	69	n.d.
Cu-ZrO_2/SiO_2[22]	4.2	230	25	3	4800	2.14	77	n.d.
Cu/ZrO_2[23]	0.8	230	25	3	2480	3.43	75	n.d.
CuZn/UiO-bpy[24]	6.9	250	40	3	4500	0.005	100	n.d.
Cu/ZrO_2[25]	37.2	220	30	3	792	0.19	58	40
Cu/a-ZrO_2[26]	9.0	230	10	3	1680	1.27	n.d.	n.d.
Cu-ZrO_2/SiO_2[27]	5.2	250	6.5	3	3600	0.98	26	58.6
$Cu/ZnO/Al_2O_3$ (JM, Katalco, this work)	41.2	250	32	3	11667	0.54	7.4	63±6
$Cu/ZnO/Al_2O_3$ (JM, Katalco)[28]	n.d.	240	124	3	1744	0.8	n.d.	n.d.
$Cu/ZnO/Al_2O_3$ (JM, Katalco)[28]	n.d.	240	96.5	3	1744	0.47	n.d.	n.d.
$Cu/ZnO/Al_2O_3$ (JM, Katalco)[29]	50	270	20	3	4500	0.65	n.d.	n.d.

[a] n.d. denotes not determined.

Note: Johnson Matthey (JM) KATALCO 51 series are the state-of-art $Cu/ZnO/Al_2O_3$ catalysts for methanol synthesis from carbon oxides and hydrogen. More information about KATALCO 51 can be found at https://matthey.com/en/products-and-services/chemical-processes/chemical-catalysts/methanol-synthesis-catalysts. The rates are normalized by the Cu loadings.

Supplementary Table 10. The signals for the fragmentation patterns, and the corresponding intensities (in brackets) for CO_2, CO, HCOOH, HCHO, and CH_3OH. These are possible major products and intermediates for CO_2 hydrogenation. The masses m/z = 44, 46, 30, and 31 are the most intense signals for CO_2, HCOOH, HCHO, and CH_3OH respectively. We did not use m/z = 28 and 32 because they can derive from several different species with relatively high intensity. Thus we did not analyze for CO.

CO_2	CO	CH_3OH	HCHO	HCOOH
44 (78.4)	28 (91.5)	31 (31.0)	29 (52.7)	29 (37.5)
28 (8.6)	12 (4.6)	29 (22.3)	30 (30.6)	46 (22.8)
16 (7.1)	16 (1.8)	32 (20.8)	28 (12.6)	45 (18.4)
12 (4.7)	29 (0.9)	15 (12.9)	15 (2.1)	17 (6.4)
45 (0.8)	14 (0.9)	28 (3.7)	14 (0.5)	28 (6.4)
46 (0.4)	30 (0.2)	14 (3.1)	13 (0.5)	44 (3.7)
		30 (2.8)	12 (0.5)	16 (1.9)
		13 (1.7)	31 (0.3)	12 (1.3)

Supplementary Table 11. Comparison of the fitting quality for Cu/UiO-66-a with a Cu–Zr path and without a Cu–Zr path. The fitting quality was greatly improved with the addition of the Cu–Zr path.

Fitting	R-factor	Chi-square
Fitting without Cu–Zr path	0.0092	107.26
Fitting with Cu–Zr path	0.0057	66.22

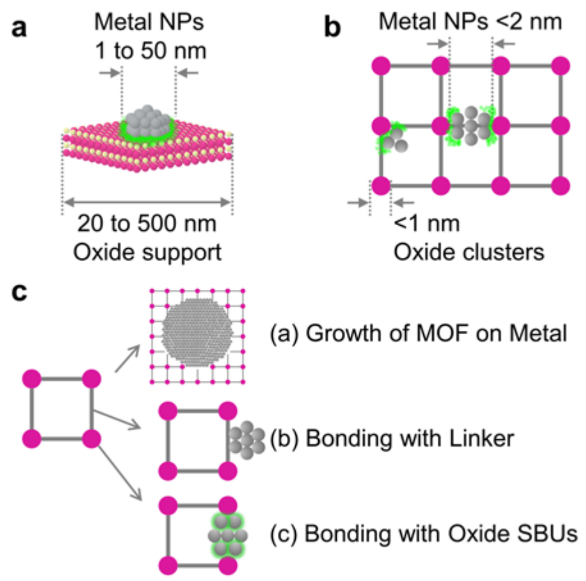

Supplementary Figure 1. Approaches for creating interfaces: (a) supported metal nanoparticles and (b) metal clusters incorporated into three-dimensional frameworks. The fraction of interfacial sites in a confined space can be maximized in the pores of an MOF, as shown in (c) which compares three situations: (a) growing MOFs around metal particles,[30] (b) grafting metal particles onto linkers,[31] and (c) anchoring metal particles onto the oxide secondary building units, as proposed in this work.

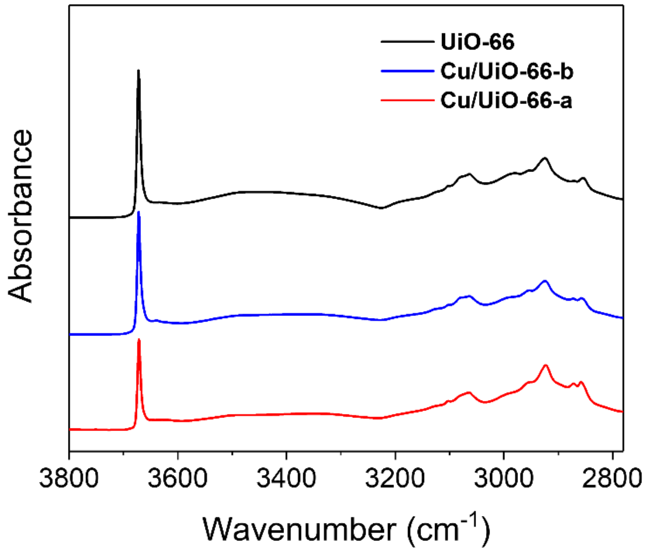

Supplementary Figure 2. Comparison of IR spectra of fresh Cu/UiO-66-a, Cu/UiO-66-b, and UiO-66 showing the decrease in intensity of OH groups (3671 cm^{-1}) on the Zr_6 nodes. The results suggest that the Cu species interact with the OH groups on the Zr_6 nodes.

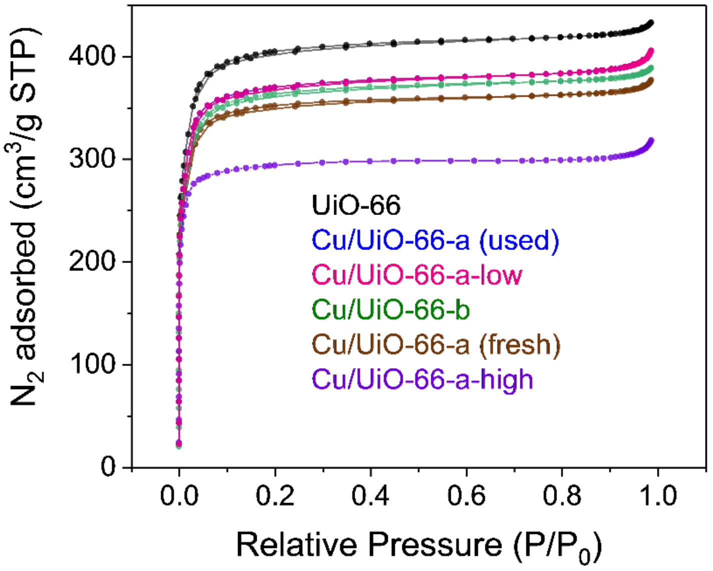

Supplementary Figure 3. The N_2-physisorption isotherms of UiO-66, fresh and used Cu/UiO-66-a, fresh Cu/UiO-66-b, Cu/UiO-66-a-low, and Cu/UiO-66-a-high. The isotherms for used Cu/UiO-66-a and Cu/UiO-66-a-low are partially overlapping.

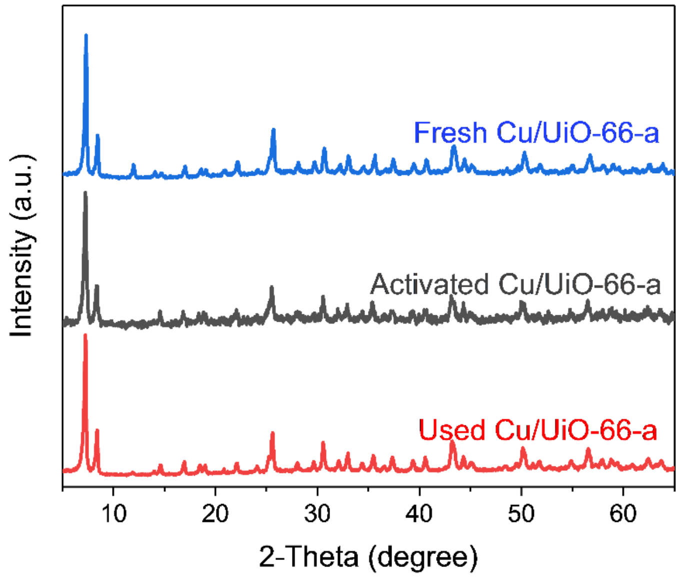

Supplementary Figure 4. XRD patterns of the fresh, activated, and used Cu/UiO-66-a.

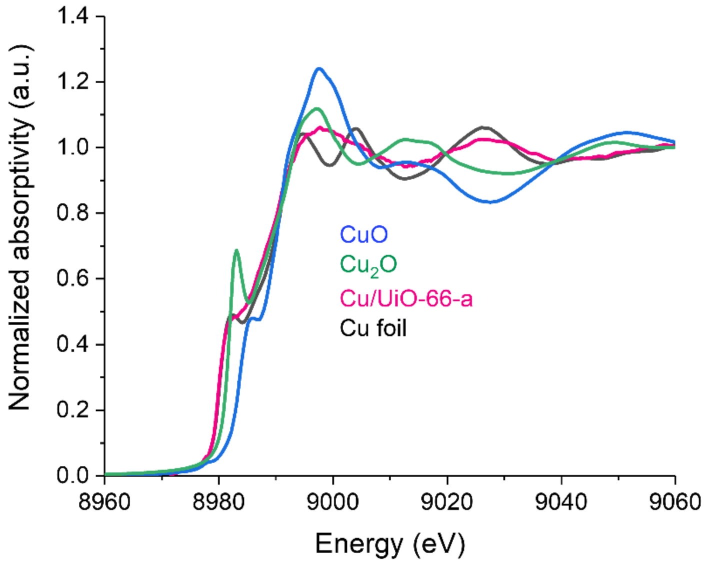

Supplementary Figure 5. XANES spectra of the activated Cu/UiO-66-a and related standards.

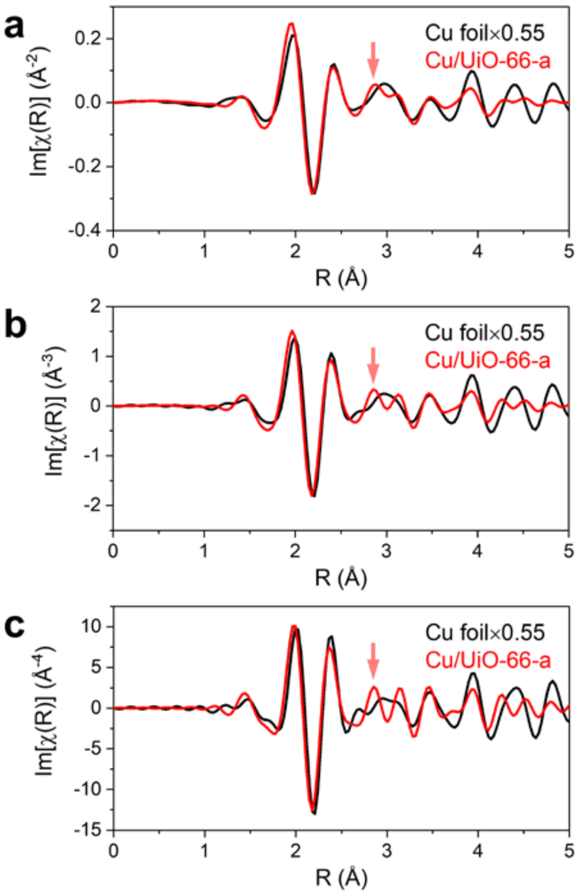

Supplementary Figure 6. Fourier-transform plots of EXAFS results of activated Cu/UiO-66-a and Cu foil with three weightings: (a) k^1 weighting, (b), k^2 weighting, (c) k^3 weighting. The Cu–Cu first shell of Cu foil has been scaled by a factor of 0.55. The results show the presence of Cu–Zr backscatter.

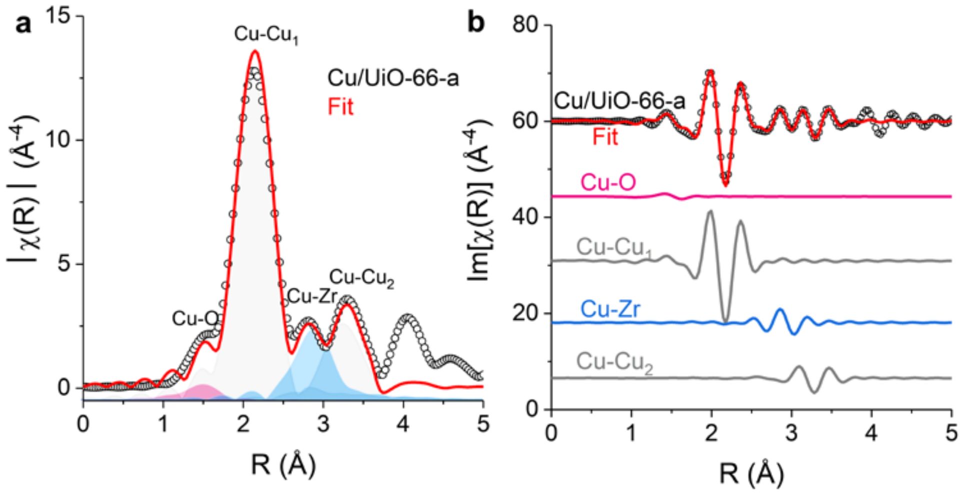

Supplementary Figure 7. Spectra for Cu/UiO-66-a. (a) Cu k-edge k^3-weighted EXAFS. (b) Fourier-transform spectra with fitting curves.

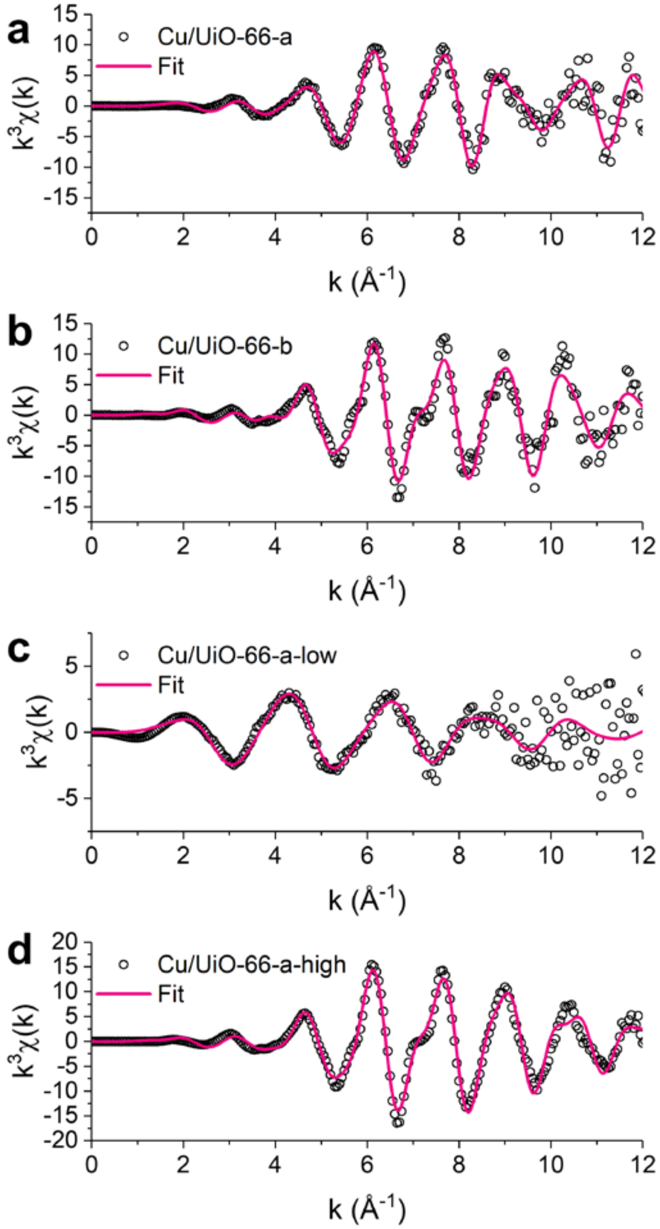

Supplementary Figure 8. The k^3-weighted experimental EXAFS oscillations of activated catalysts and the corresponding simulation results: (a) Cu/UiO-66-a; (b) Cu/UiO-66-b; (c) Cu/CuiO-66-a-low; and (d) Cu/UiO-66-a-high.

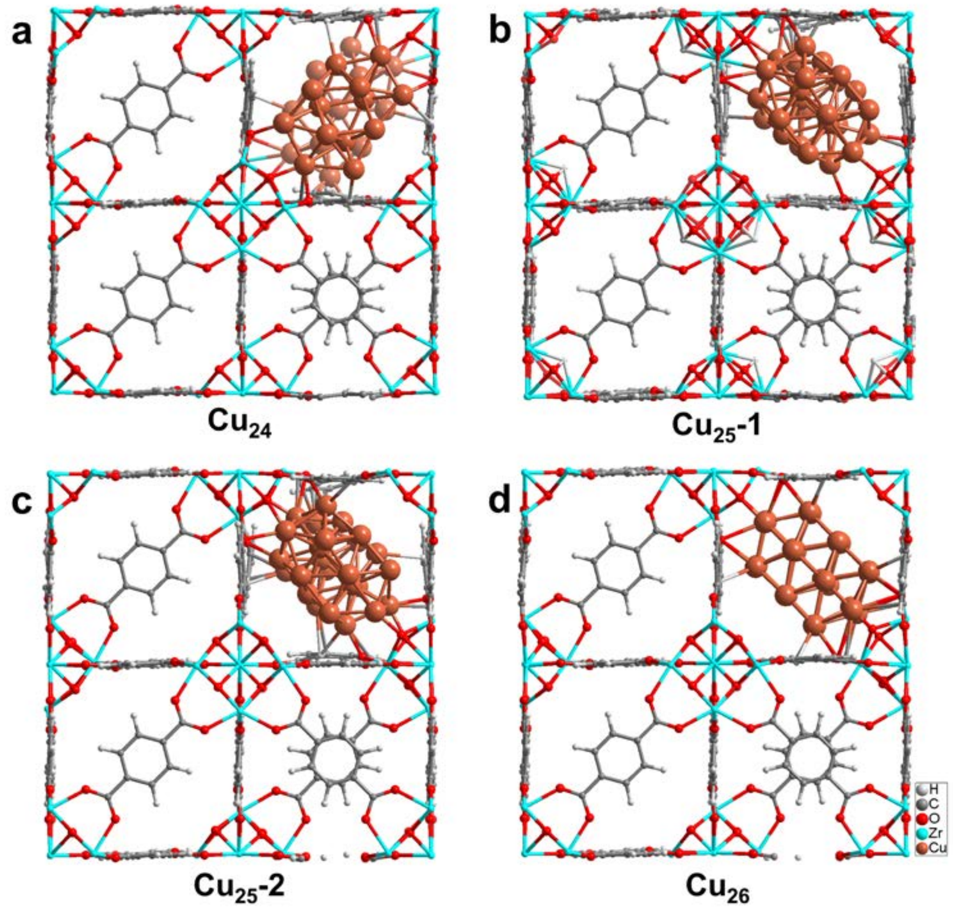

Supplementary Figure 9. Density-functional-optimized structures of Cu clusters stabilized in the defective pores of UiO-66. The Cu clusters in this figure contain: (a) 24 Cu atoms; (b) 24 Cu atoms (configuration 1); (c) 25 Cu atoms (configuration 2); and (d) 26 Cu atoms.

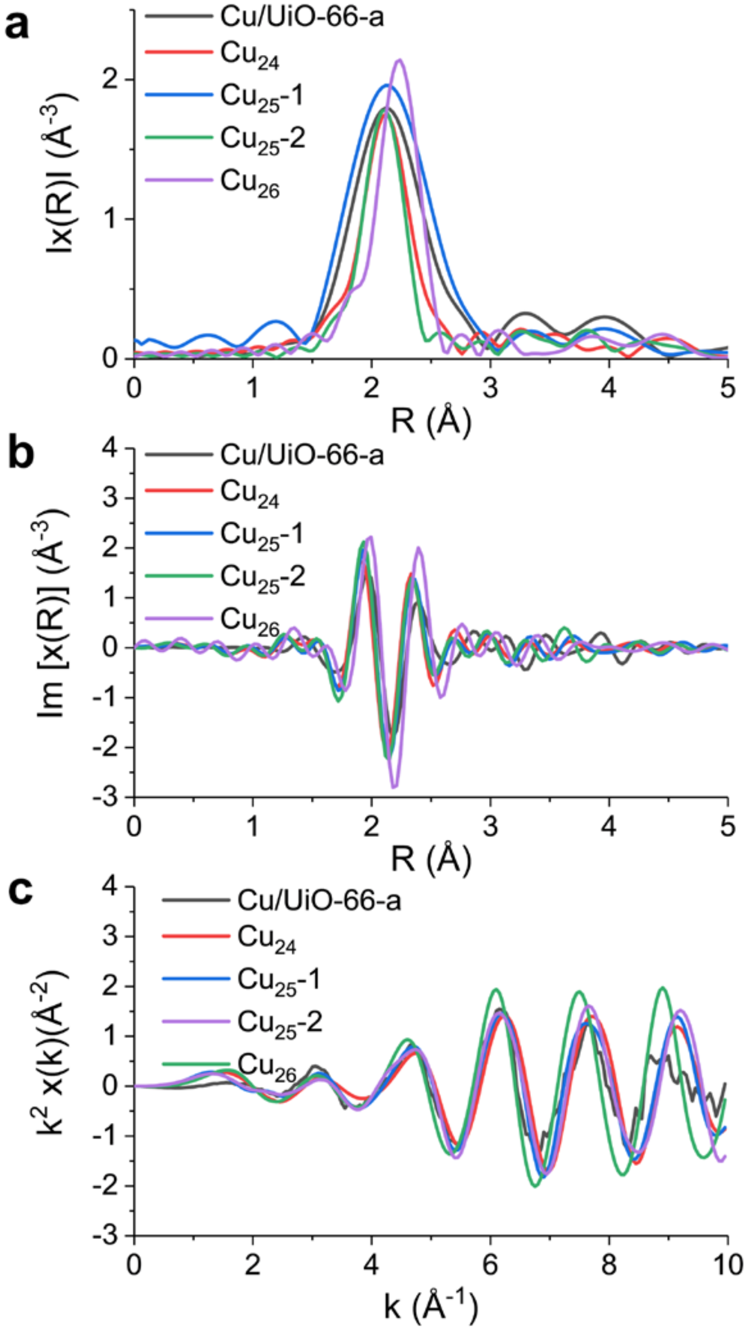

Supplementary Figure 10. The k^2-weighted Cu-EXAFS spectra of experimentally measured Cu/UiO-66-a (black curve) and calculated EXAFS spectra of density-functional-optimized Cu clusters in the pore of UiO-66 MOF. (a) |χ(R)|. (b) x(k). (c) Img[χ(R)]. The structures of the optimized Cu clusters can be found in Supplementary Figure 9. The spectra corresponding to the Cu_{25}-1 structure match the traces of the experimental spectra in x(k) and x(R) plots better than the spectra corresponding to the other structures. The average Cu-O, Cu–Zr, and $Cu-Cu_1$ distances in the Cu_{25}-1 structure are 2.04, 3.09, and 2.56 Å, respectively, and these are close to the distances inferred from the EXAFS fitting.

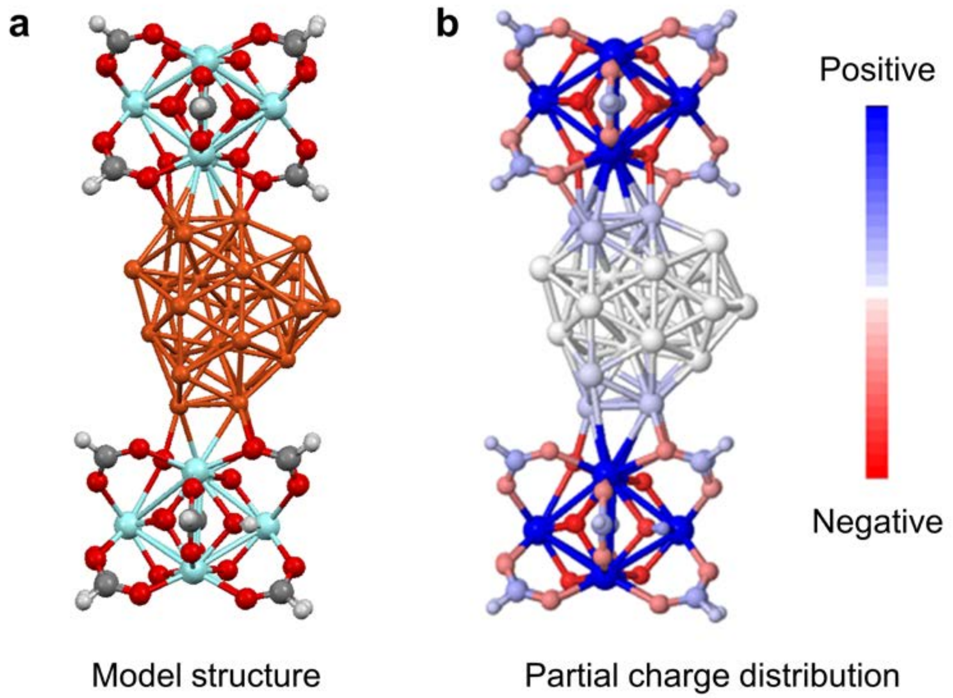

Supplementary Figure 11. Simplified model structure of Cu/UiO-66-a. This figure illustrates the density functional results in Table S3. (a) Colors denote elements (Cu, H, O, and Zr). (b) Colors denote CM5 partial atomic charges.

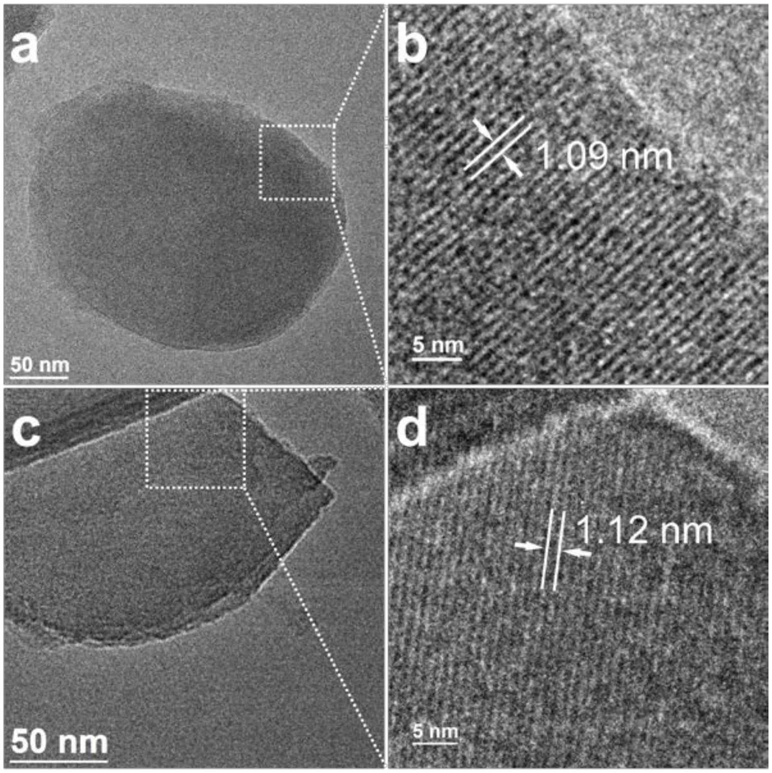

Supplementary Figure 12. HRTEM images of Cu/UiO-66-a. (a) Particle 1 with scale of 50 nm; (b) particle 1 with scale of 5 nm; (c) particle 2 with scale of 50 nm; and (d) particle 2 with scale of 5 nm.

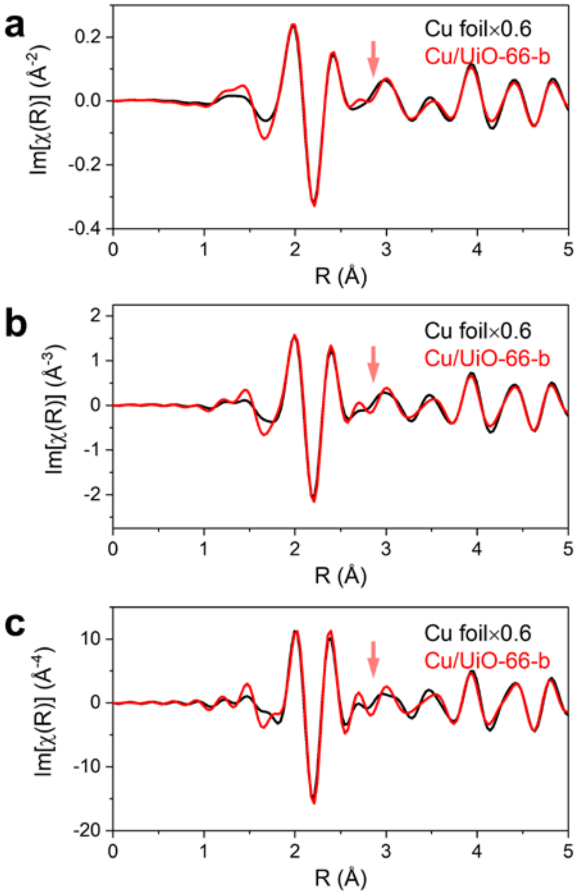

Supplementary Figure 13. Fourier-transform plots of EXAFS results of activated Cu/UiO-66-b and Cu foil. (a) k^1 weighting. (b), k^2 weighting, (c) k^3 weighting. The Cu–Cu first shell of Cu foil has been scaled by a factor of 0.55. The results indicate the absence of Cu–Zr backscatter.

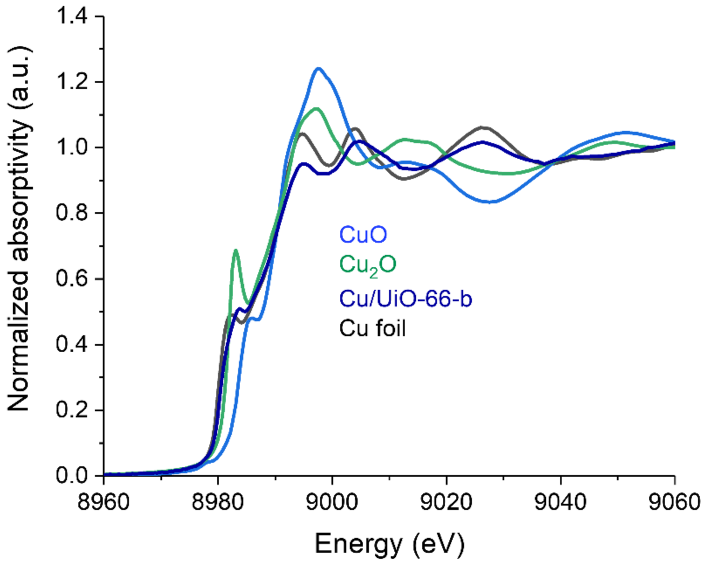

Supplementary Figure 14. XANES spectra of the activated Cu/UiO-66-b and related standards.

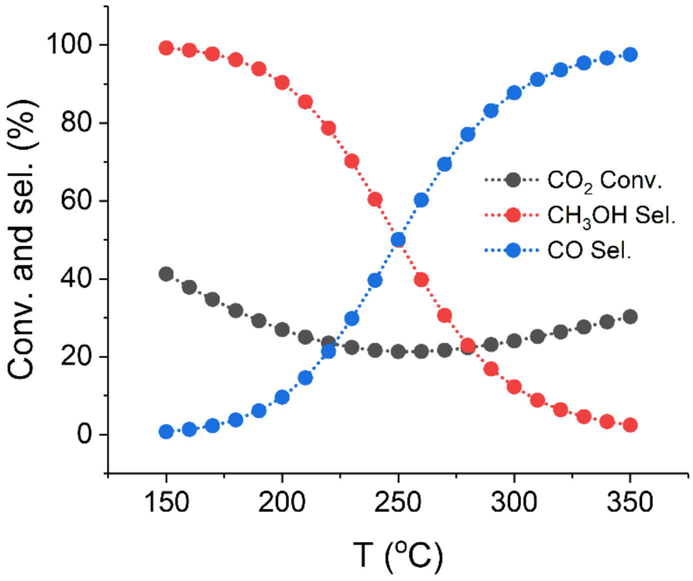

Supplementary Figure 15. Effect of reaction temperature on the thermodynamic equilibrium of CO_2 conversion and on product selectivity for CO and methanol products at 32 bar with an inlet CO_2:H_2 ratio of 1:3.

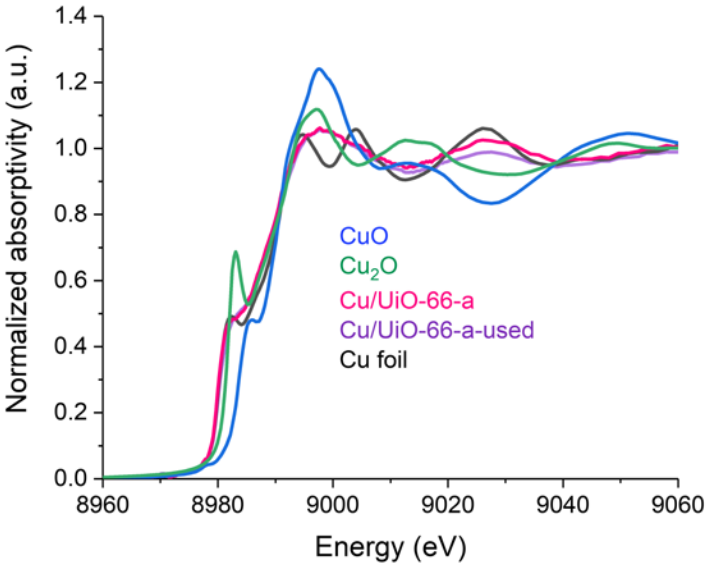

Supplementary Figure 16. XANES spectra of the activated and used Cu/UiO-66-a as well as two reference materials.

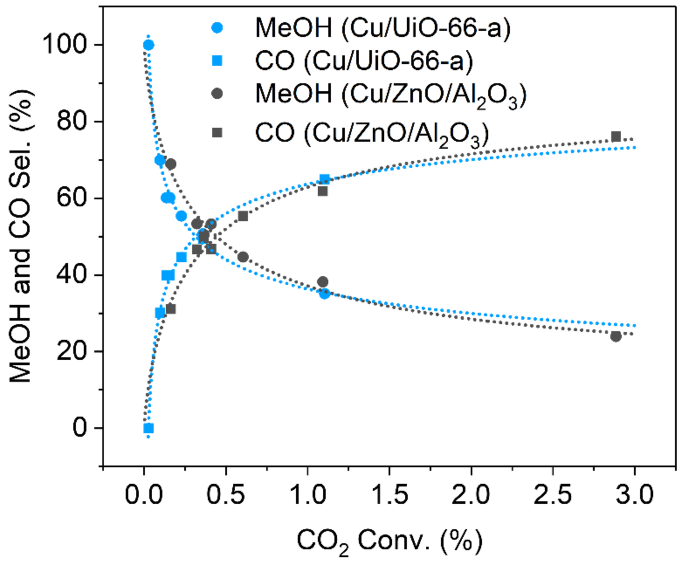

Supplementary Figure 17. Selectivity to methanol and CO as a functions of CO_2 conversion for Cu/UiO-66-a and Cu/ZnO/Al_2O_3 at 190ºC and 32 bar. The CO_2 conversion was varied by changing the gas space velocity.

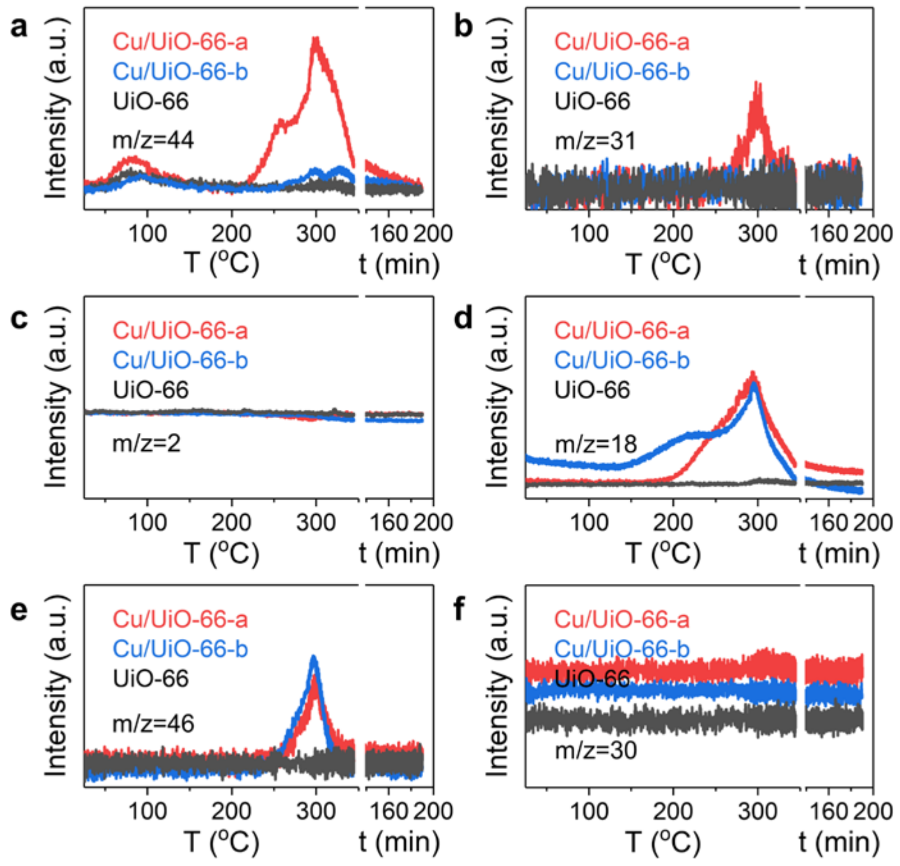

Supplementary Figure 18. Evolution of different fragments monitored during temperature-programmed desorption after CO_2 adsorption. Red, blue, and black traces correspond to desorption from Cu/UiO-66-a, Cu/UiO-66-b, and UiO-66, respectively. The selected fragments are attributed to: CO_2 (m/z = 44, a), CH_3OH (m/z = 31, b), H_2 (m/z = 2, c), H_2O (m/z = 18, d), HCOOH (m/z = 46, e), and HCHO (m/z = 30, f). The assignments are given in **Table S10**. Prior to desorption, the materials were exposed to 8 bar CO_2 at 25°C. The signal of H_2 was used to normalize the profiles. The left part of figures is the ramping process from 25 to 350°C while the right part of figures is the process at the constant temperature of 350°C.

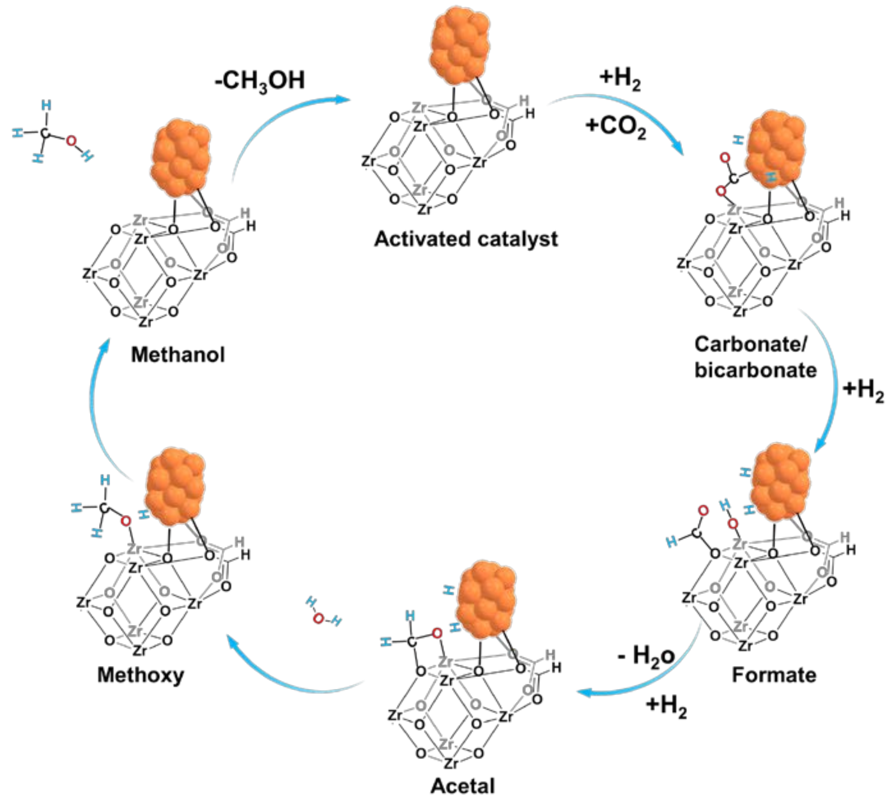

Supplementary Figure 19. Catalytic cycle for the conversion of CO_2 to methanol at the Cu-Zr_6 nodes interface.

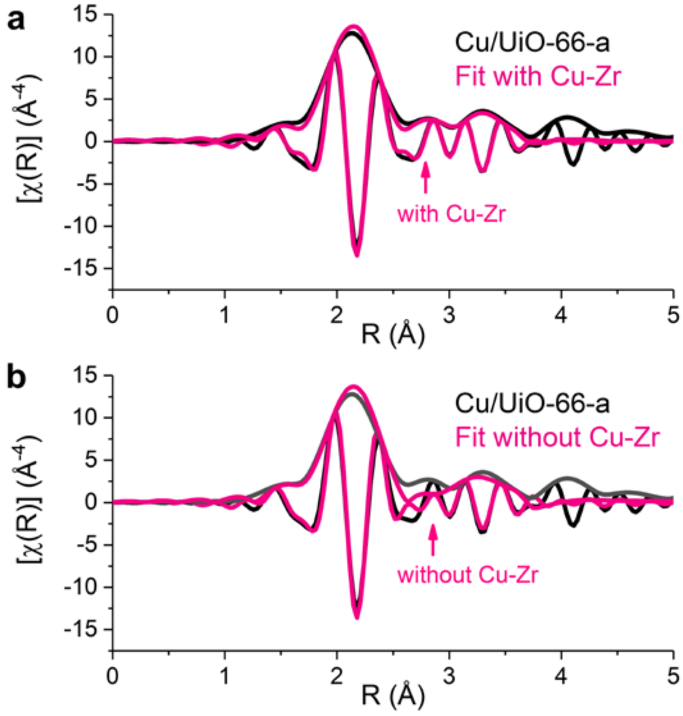

Supplementary Figure 20. The EXAFS fitting spectra for Cu/UiO-66-a. (a) With Cu–Zr path. (b) Without Cu–Zr path. The R-factor and Chi-square of the fittings can be found in Supplementary Table 11.

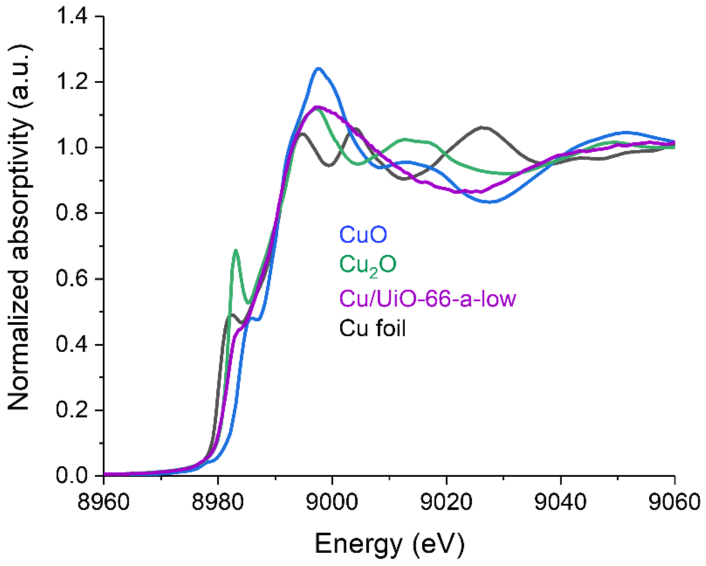

Supplementary Figure 21. XANES spectra of activated Cu/UiO-66-a-low and related standards.

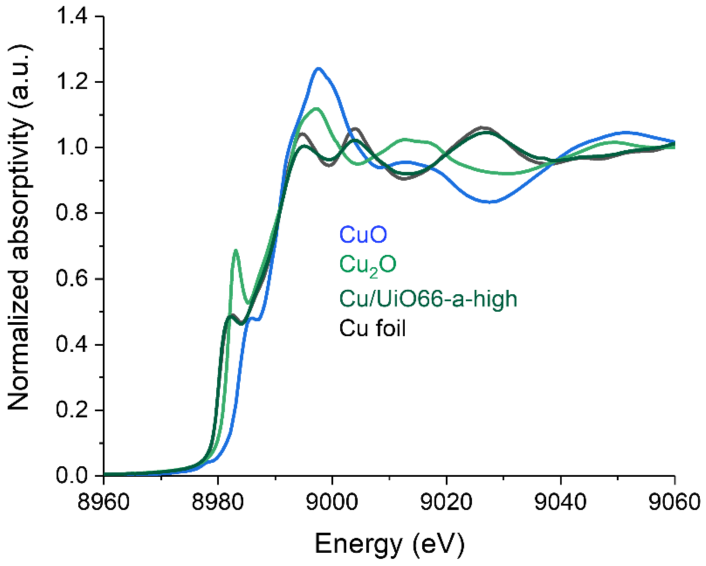

Supplementary Figure 22. XANES spectra of the activated Cu/UiO-66-a-high and related standards.

Supplementary References

1. Abdel-Mageed AM, Rungtaweevoranit B, Parlinska-Wojtan M, Pei X, Yaghi OM, Behm RJ. Highly Active and Stable Single-Atom Cu Catalysts Supported by a Metal–Organic Framework. *J Am Chem Soc* **141**, 5201-5210 (2019).

2. Wu H, *et al.* Unusual and Highly Tunable Missing-Linker Defects in Zirconium Metal–Organic Framework UiO-66 and Their Important Effects on Gas Adsorption. *J Am Chem Soc* **135**, 10525-10532 (2013).

3. Katz MJ, *et al.* A facile synthesis of UiO-66, UiO-67 and their derivatives. *Chem Commun* **49**, 9449-9451 (2013).

4. Nguyen HGT, *et al.* Vanadium-Node-Functionalized UiO-66: A Thermally Stable MOF-Supported Catalyst for the Gas-Phase Oxidative Dehydrogenation of Cyclohexene. *ACS Catal* **4**, 2496-2500 (2014).

5. Mensah JB, Delidovich I, Hausoul PJC, Weisgerber L, Schrader W, Palkovits R. Mechanistic Studies of the $Cu(OH)^+$-Catalyzed Isomerization of Glucose into Fructose in Water. *ChemSusChem* **11**, 2579-2586 (2018).

6. Grundner S, *et al.* Single-site trinuclear copper oxygen clusters in mordenite for selective conversion of methane to methanol. *Nat Commun* **6**, 7546 (2015).

7. Ren M, Zhou Y, Tao F, Zou Z, Akins DL, Yang H. Controllable Modification of the Electronic Structure of Carbon-Supported Core–Shell Cu@Pd Catalysts for Formic Acid Oxidation. *J Phys Chem C* **118**, 12669-12675 (2014).

8. Jentys A. Estimation of mean size and shape of small metal particles by EXAFS. *Phys Chem Chem Phys* **1**, 4059-4063 (1999).

9. Kip BJ, Duivenvoorden FBM, Koningsberger DC, Prins R. Determination of metal particle size of highly dispersed Rh, Ir, and Pt catalysts by hydrogen chemisorption and EXAFS. *J Catal* **105**, 26-38 (1987).

10. Ikuno T, *et al.* Methane Oxidation to Methanol Catalyzed by Cu-Oxo Clusters Stabilized in NU-1000 Metal–Organic Framework. *J Am Chem Soc* **139**, 10294-10301 (2017).

11. Hutter J, Iannuzzi M, Schiffmann F, VandeVondele J. cp2k: atomistic simulations of condensed matter systems. *WIREs Computational Molecular Science* **4**, 15-25 (2014).

12. Perdew JP, Burke K, Ernzerhof M. Generalized Gradient Approximation Made Simple. *Phys Rev Lett* **77**, 3865-3868 (1996).

13. Grimme S, Antony J, Ehrlich S, Krieg H. A consistent and accurate ab initio parametrization of density functional dispersion correction (DFT-D) for the 94 elements H-Pu. *J Chem Phys* **132**, 154104 (2010).

14. Goedecker S, Teter M, Hutter J. Separable dual-space Gaussian pseudopotentials. *Phys Rev B* **54**, 1703-1710 (1996).

15. Marenich AV, Jerome SV, Cramer CJ, Truhlar DG. Charge Model 5: An Extension of Hirshfeld Population Analysis for the Accurate Description of Molecular Interactions in Gaseous and Condensed Phases. *J Chem Theory Comput* **8**, 527-541 (2012).

16. Koningsberger DC, Mojet BL, van Dorssen GE, Ramaker DE. XAFS spectroscopy; fundamental principles and data analysis. *Top Catal* **10**, 143-155 (2000).

17. Grundner S, *et al.* Single-site trinuclear copper oxygen clusters in mordenite for selective conversion of methane to methanol. *Nat Commun* **6**, 7546 (2015).

18. Zhu Y, *et al.* Inverse iron oxide/metal catalysts from galvanic replacement. *Nat Commun* **11**, 3269 (2020).

19. Kattel S, Yan B, Yang Y, Chen JG, Liu P. Optimizing Binding Energies of Key Intermediates for CO_2 Hydrogenation to Methanol over Oxide-Supported Copper. *J Am Chem Soc* **138**, 12440-12450 (2016).

20. Natesakhawat S, *et al.* Active Sites and Structure–Activity Relationships of Copper-Based Catalysts for Carbon Dioxide Hydrogenation to Methanol. *ACS Catal* **2**, 1667-1676 (2012).

21. Witoon T, Chalorngtham J, Dumrongbunditkul P, Chareonpanich M, Limtrakul J. CO_2 hydrogenation to methanol over Cu/ZrO_2 catalysts: Effects of zirconia phases. *Chem Eng J* **293**, 327-336 (2016).

22. Lam E, Larmier K, Wolf P, Tada S, Safonova OV, Copéret C. Isolated Zr Surface Sites on Silica Promote Hydrogenation of CO_2 to CH_3OH in Supported Cu Catalysts. *J Am Chem Soc* **140**, 10530-10535 (2018).

23. Larmier K, *et al.* CO_2-to-Methanol Hydrogenation on Zirconia-Supported Copper Nanoparticles: Reaction Intermediates and the Role of the Metal-Support Interface. *Angew Chem Int Ed* **56**, 2318-2323 (2017).

24. An B, Zhang J, Cheng K, Ji P, Wang C, Lin W. Confinement of Ultrasmall Cu/ZnO_x Nanoparticles in Metal–Organic Frameworks for Selective Methanol Synthesis from Catalytic Hydrogenation of CO_2. *J Am Chem Soc* **139**, 3834-3840 (2017).

25. Guo X, Mao D, Lu G, Wang S, Wu G. The influence of La doping on the catalytic behavior of Cu/ZrO_2 for methanol synthesis from CO_2 hydrogenation. *J Mol Catal A: Chem* **345**, 60-68 (2011).

26. Tada S, *et al.* Effects of Cu Precursor Types on the Catalytic Activity of Cu/ZrO_2 toward Methanol Synthesis via CO_2 Hydrogenation. *Ind Eng Chem Res* **58**, 19434-19445 (2019).

27. Schilke TC, Fisher IA, Bell AT. Influence of titania on zirconia promoted Cu/SiO_2 catalysts for methanol synthesis from CO/H_2 and CO_2/H_2. *Catal Lett* **54**, 105-111 (1998).

28. Doss B, Ramos C, Atkins S. Optimization of Methanol Synthesis from Carbon Dioxide and Hydrogen: Demonstration of a Pilot-Scale Carbon-Neutral Synthetic Fuels Process. *Energy & Fuels* **23**, 4647-4650 (2009).

29. Tada S, Larmier K, Büchel R, Copéret C. Methanol synthesis via CO_2 hydrogenation over CuO–ZrO_2 prepared by two-nozzle flame spray pyrolysis. *Catal Sci Technol* **8**, 2056-2060 (2018).

30. Rungtaweevoranit B, *et al.* Copper Nanocrystals Encapsulated in Zr-based Metal-Organic Frameworks for Highly Selective CO_2 Hydrogenation to Methanol. *Nano Lett* **16**, 7645-7649 (2016).

31. An B, Zhang J, Cheng K, Ji P, Wang C, Lin W. Confinement of Ultrasmall Cu/ZnO_x Nanoparticles in Metal-Organic Frameworks for Selective Methanol Synthesis from Catalytic Hydrogenation of CO_2. *J Am Chem Soc* **139**, 3834-3840 (2017).
