# Supplementary material for: Copper-zirconia interfaces in UiO-66 enable selective catalytic hydrogenation of CO2 to methanol
Source: Nat Commun. 2020 Nov 18;11:5849. doi: 10.1038/s41467-020-19438-w (PMC7674450; doi:10.1038/s41467-020-19438-w)
Supplement: Supplementary file 2 — Description of Additional Supplementary Files [file 41467_2020_19438_MOESM2_ESM.pdf]

**Description of Additional Supplementary Files**

File Name: Supplementary Data 1
Description: Cartesian coordinates and calculated partial atomic charges of the model of Cu/UiO-66-a as optimized by density functional calculations.
