# Supplementary material for: Copper-zirconia interfaces in UiO-66 enable selective catalytic hydrogenation of CO2 to methanol
Source: Nat Commun. 2020 Nov 18;11:5849. doi: 10.1038/s41467-020-19438-w (PMC7674450; doi:10.1038/s41467-020-19438-w)
Supplement: Supplementary file 3 — Supplementary Data 1 [file 41467_2020_19438_MOESM3_ESM.pdf]

Cartesian coordinates of the model of Cu/UiO-66-a as optimized by density functional calculations. The *x*, *y*, and *z* columns are in Å. The last column contains the calculated partial atomic charges. The model structure and charge distribution are shown in Supplementary Figure 10.

No.	Element	*x*	*y*	*z*	Charge
1	C	20.86972	7.317925	13.624	0.241
2	C	10.42975	17.7085	13.64717	0.225
3	C	20.76713	13.61349	7.211587	0.216
4	C	10.68373	17.78743	7.20264	0.210
5	C	7.256031	20.85433	13.62721	0.241
6	C	17.71576	10.6244	7.220057	0.227
7	C	17.66328	7.338099	10.56606	0.233
8	C	7.290449	17.70309	10.55654	0.227
9	C	20.78667	13.56136	13.66199	0.235
10	C	21.00609	7.19042	7.390976	0.24
11	C	24.16418	10.48116	13.55949	0.242
12	C	24.1243	10.29247	7.342596	0.233
13	C	23.99577	13.68144	10.56044	0.229
14	C	10.45092	24.12146	13.53772	0.234
15	C	10.40815	24.0374	7.274119	0.242
16	C	13.61763	20.83943	13.63589	0.230
17	C	7.215095	21.03286	7.340245	0.225
18	C	13.63595	24.01878	10.50706	0.233
19	H	8.587006	18.95556	8.472026	0.347
20	H	22.84656	12.37945	8.490693	0.341
21	H	10.47638	16.87192	14.4202	0.114
22	H	10.82523	17.04694	6.347505	0.103
23	H	6.413839	20.8561	14.39552	0.119
24	H	10.47487	24.98422	14.28248	0.114
25	H	10.40992	24.89646	6.524702	0.120
26	H	14.45353	20.75791	14.40675	0.113
27	H	6.361006	21.08712	6.587127	0.115
28	H	20.8456	6.467679	14.38301	0.120
29	H	20.69879	14.36527	6.357323	0.120
30	H	16.88639	10.65945	6.4387	0.115
31	H	20.99348	6.386161	6.583134	0.119
32	H	25.00607	10.4734	14.32809	0.121
33	H	24.97963	10.2336	6.591236	0.118
34	H	20.75303	14.34602	14.48829	0.119
35	H	16.8812	6.508711	10.55798	0.116
36	H	6.505493	16.87908	10.62331	0.116
37	H	24.7523	14.53163	10.62717	0.118
38	H	14.39906	24.8656	10.4942	0.116

No.	Element	*x*	*y*	*z*	Charge
39	O	20.71472	14.02942	8.461392	-0.395
40	O	10.60459	17.36095	8.42071	-0.372
41	O	10.44597	17.29532	12.42499	-0.381
42	O	20.95957	6.941071	12.4061	-0.357
43	O	8.475061	20.8167	14.01426	-0.359
44	O	18.92643	10.51915	6.855761	-0.338
45	O	17.29325	8.573091	10.60607	-0.382
46	O	6.929069	18.9295	10.5253	-0.375
47	O	17.30765	10.70014	8.463022	-0.392
48	O	6.855902	20.89229	12.40508	-0.380
49	O	10.36825	18.9267	14.02719	-0.353
50	O	20.80743	8.549878	14.02742	-0.388
51	O	10.61512	19.02184	6.846717	-0.370
52	O	20.89187	12.40296	6.851177	-0.338
53	O	8.503924	17.29434	10.51826	-0.371
54	O	18.87655	6.941582	10.53326	-0.3634
55	O	19.24749	11.92136	11.65155	-0.706
56	O	11.8407	19.43277	11.73446	-0.729
57	O	19.58542	9.14586	9.14109	-0.777
58	O	9.132451	19.50189	9.061718	-0.690
59	O	19.82621	11.60537	9.245254	-0.794
60	O	19.67594	9.387774	11.69473	-0.788
61	O	9.382002	19.75827	11.51429	-0.794
62	O	11.75394	19.65203	9.211323	-0.746
63	O	21.05681	6.85425	8.634419	-0.373
64	O	20.80457	14.02616	12.46766	-0.367
65	O	22.94346	10.52742	13.9467	-0.369
66	O	22.91855	10.36386	6.932168	-0.360
67	O	24.39879	12.4678	10.52671	-0.371
68	O	24.49073	10.27955	8.580308	-0.387
69	O	24.55485	10.44066	12.33295	-0.374
70	O	20.96635	8.393874	6.944464	-0.369
71	O	20.80395	12.31163	13.96268	-0.374
72	O	22.76677	14.0474	10.52466	-0.376
73	O	22.27259	9.132652	11.73081	-0.759
74	O	22.27422	11.83616	9.056148	-0.681
75	O	22.10094	9.303492	9.304912	-0.784
76	O	21.94448	11.60055	11.50601	-0.807
77	O	10.28262	24.39621	8.495583	-0.361
78	O	10.43451	24.48467	12.30828	-0.378
79	O	8.424799	20.96155	6.931213	-0.369
80	O	12.40647	20.95246	14.01832	-0.341

No.	Element	*x*	*y*	*z*	Charge
81	O	14.03352	22.79347	10.51079	-0.379
82	O	6.846961	21.05041	8.575171	-0.380
83	O	14.02995	20.80468	12.4062	-0.383
84	O	10.44252	22.91411	13.95814	-0.362
85	O	10.52725	22.81767	6.860111	-0.382
86	O	12.4139	24.39443	10.51682	-0.367
87	O	9.056911	22.26799	11.6655	-0.748
88	O	11.75967	22.11026	9.134935	-0.773
89	O	9.22881	22.03237	9.241918	-0.789
90	O	11.59051	22.02211	11.67078	-0.807
91	Zr	10.49239	18.38351	10.46644	1.302
92	Zr	18.51342	10.43847	10.33159	1.359
93	Zr	20.8188	12.94795	10.47249	1.333
94	Zr	20.79536	8.124967	10.46736	1.538
95	Zr	21.03915	10.3823	12.85825	1.561
96	Zr	20.90537	10.36443	7.962841	1.532
97	Zr	23.31622	10.49164	10.47614	1.574
98	Zr	10.50243	23.23596	10.44446	1.552
99	Zr	10.4094	20.90677	12.92937	1.516
100	Zr	10.42391	20.96697	8.034251	1.545
101	Zr	12.81489	20.92206	10.51781	1.293
102	Zr	8.035123	20.87596	10.52363	1.563
124	Cu	13.98693	14.30843	9.488294	-0.016
105	Cu	15.14195	15.61608	5.829664	-0.014
113	Cu	14.87709	13.65698	7.312814	-0.012
122	Cu	16.04004	18.32621	8.92934	-0.0101
109	Cu	15.14401	17.34353	12.85225	-0.010
123	Cu	17.91066	16.67734	8.946549	-0.008
121	Cu	17.0786	17.50465	11.14786	-0.008
118	Cu	16.93741	14.80838	7.374937	-0.007
126	Cu	14.88987	16.7044	10.48478	0.00009
127	Cu	14.89312	16.19418	8.12802	0.002
110	Cu	14.62765	18.24894	6.887407	0.003
112	Cu	15.29979	13.24331	11.21991	0.003
108	Cu	16.82442	17.18954	6.825117	0.007
125	Cu	16.45153	14.89464	9.82249	0.008
117	Cu	13.73428	15.14252	11.80386	0.0151
119	Cu	16.28507	15.31044	12.19872	0.015
115	Cu	12.92621	15.15471	7.215502	0.029
107	Cu	18.56816	15.54917	11.06959	0.046
114	Cu	12.60375	16.27456	9.59222	0.066
106	Cu	15.11929	19.13998	11.07342	0.099

No.	Element	*x*	*y*	*z*	Charge
116	Cu	16.51387	12.67458	9.003979	0.102
120	Cu	13.595	18.49155	9.10347	0.117
111	Cu	18.65233	14.24367	9.00268	0.142
103	Cu	17.74857	13.17384	11.14947	0.145
104	Cu	13.0157	17.75282	11.56242	0.182
